# The *Trypanosoma cruzi *Sylvio X10 strain maxicircle sequence: the third musketeer

**DOI:** 10.1186/1471-2164-12-58

**Published:** 2011-01-24

**Authors:** Laura I Ruvalcaba-Trejo, Nancy R Sturm

**Affiliations:** 1 Department of Microbiology, Immunology & Molecular Genetics, David Geffen School of Medicine, University of California at Los Angeles, Los Angeles, CA 90095-1489, USA

## Abstract

**Background:**

Chagas disease has a diverse pathology caused by the parasite *Trypanosoma cruzi*, and is indigenous to Central and South America. A pronounced feature of the trypanosomes is the kinetoplast, which is comprised of catenated maxicircles and minicircles that provide the transcripts involved in uridine insertion/deletion RNA editing. *T. cruzi *exchange genetic material through a hybridization event. Extant strains are grouped into six discrete typing units by nuclear markers, and three clades, A, B, and C, based on maxicircle gene analysis. Clades A and B are the more closely related. Representative clade B and C maxicircles are known in their entirety, and portions of A, B, and C clades from multiple strains show intra-strain heterogeneity with the potential for maxicircle taxonomic markers that may correlate with clinical presentation.

**Results:**

To perform a genome-wide analysis of the three maxicircle clades, the coding region of clade A representative strain Sylvio X10 (a.k.a. Silvio X10) was sequenced by PCR amplification of specific fragments followed by assembly and comparison with the known CL Brener and Esmeraldo maxicircle sequences. The clade A rRNA and protein coding region maintained synteny with clades B and C. Amino acid analysis of non-edited and 5'-edited genes for Sylvio X10 showed the anticipated gene sequences, with notable frameshifts in the non-edited regions of Cyb and ND4. Comparisons of genes that undergo extensive uridine insertion and deletion display a high number of insertion/deletion mutations that are likely permissible due to the post-transcriptional activity of RNA editing.

**Conclusion:**

Phylogenetic analysis of the entire maxicircle coding region supports the closer evolutionary relationship of clade B to A, consistent with uniparental mitochondrial inheritance from a discrete typing unit TcI parental strain and studies on smaller fragments of the mitochondrial genome. Gene variance that can be corrected by RNA editing hints at an unusual depth for maxicircle taxonomic markers, which will aid in the ability to distinguish strains, their corresponding symptoms, and further our understanding of the *T. cruzi *population structure. The prevalence of apparently compromised coding regions outside of normally edited regions hints at undescribed but active mechanisms of genetic exchange.

## Background

*Trypanosoma cruzi *is a unicellular eukaryotic organism that causes a deadly condition referred to as Chagas disease indigenous to Central and South America. Approximately 18-20 million people are infected with *T. cruzi*, with between 30,000 and 50,000 deaths per year due to chronic Chagas disease. Thirty-percent of patients infected show chronic stage Chagas disease symptoms, including mega syndromes such as enlargement of the esophagus, colon or heart, or complications in the nervous system, ultimately causing death [1,2]. Transmission of *T. cruzi *to humans occurs via a blood-sucking vector, the triatomine bug, a.k.a. the assassin bug, which is part of the Reduviidae subfamily. As the triatomine takes a blood meal from the human host, the bug leaves behind *T. cruzi*-contaminated feces on the skin of the host. Scratching of the wound causes self-infection, as *T. cruzi *enters the blood stream and replicates in macrophages, but eventually settles in smooth and cardiac tissues.

The members of the Order Trypanosomatida have a distinguishing factor: the kinetoplast. The kinetoplast is a disk-like structure that contains mitochondrial DNA (kDNA) in the form of dozens of maxicircles (20-40 kb) and thousands of minicircles (0.5-10 kb) in a catenated network with varying sizes depending on species [3]. The astounding process known as uridine insertion/deletion RNA editing produces the dramatic alterations required to convert skeletal primary transcripts into functional messages through the post-transcriptional addition of up to half of the coding information. The editing process is directed by the largely minicircle-coded guide RNAs (gRNAs) that specify the insertion or deletion of uridines within some of the maxicircle-encoded transcripts [4].

The population structure of *T. cruzi *has been of great interest in the quest to link parasite genetics to clinical disease. The non-meiotic exchange of genetic information [5] between the major lineages may have occurred only twice in the entire history of the species [6,7], although more regular exchange among close relatives may provide a unique form of intra-strain copy correction [7-10]. Currently six classes of *T. cruzi *groups are defined, termed discrete typing units (DTUs) [11,12] and referred to as *T. cruzi *I-VI, or TcI-VI [13]; in this updated nomenclature, TcI is the equivalent of DTU I, TcII of DTU IIb, and TcIII-VI of DTUs IIc, IIa, IId and IIe, respectively.

Kinetoplast biology may play a direct role in disease and pathogenesis [8]. Minicircles can integrate into the host genome [1,14,15], with the potential to effect gene expression in host cells and trigger an autoimmune response. Maxicircle gene deletions were associated with asymptomatic patients infected with *T. cruzi *[16], although the correlation was not sustained [10]. Both minicircles and maxicircles have been used as taxonomic markers [17-19] in the effort to correlate specific strains with clinical manifestations, although the usefulness of the heterogeneous minicircles is debatable [20].

The genealogical relationship of 45 *T. cruzi *strains was constructed using the 1.5-kb maxicircle COII-NDI region, defining three clades, A, B and C, in which A and B form a monophyletic group, with a basal clade C [18]. The analysis of mitochondrial gene Cyb in 20 *T. cruzi *strains also showed a similar pattern of clades and clade relationship [21]. The CL Brener and Esmeraldo strains, members of DTUs TcVI and TcII, respectively, were the focus for whole genome shotgun sequencing [22]. Using the sequence reads generated from both strains, complete maxicircle sequences for both Esmeraldo and CL Brener were assembled [23] and the minicircle components analyzed [24]. The CL Brener maxicircle is a representative of mitochondrial clade B, which includes strains from DTUs TcIII-VI, and the Esmeraldo maxicircle a mitochondrial clade C exemplar. Complete open reading frames (ORFs), partial ORFs, frame shifts, and genes with missing start codons are representative of the mitochondrial genes found on the maxicircle coding region, most of which are corrected at the transcript level by the RNA editing process. Strikingly, both of the *T. cruzi *maxicircles contain frameshifts in areas not usually subject to RNA editing, with Esmeraldo carrying a substantial deletion encompassing the 5' end of two opposing genes. These degenerations were attributed to the time the strains spent in laboratory culture, however the finding of the Esmeraldo deletion in the human population [10] indicates that maxicircle deletions are a normal part of maxicircle biology. Speculation has begun as to how the cells survive with partial mitochondrial function due to the lack of Complex I [25]; the bioenergetic consequences are minimal in *T. cruzi *strains with compromised maxicircle genes [26], consistent with survival of experimentally compromised *T. brucei *lines that demonstrate Complex I participation in electron transport [27].

To further examine *T. cruzi *maxicircles and better understand their genetic variation, we amplified and sequenced the coding region of a clade A/DTU TcI representative Sylvio X10 to compare and contrast gene synteny and content. Comparison of non-edited genes and 5'-edited genes showed functional genes in Sylvio X10 and CL Brener that are mutated in Esmeraldo. Analysis of the three clades showed strain specific insertion/deletion mutations and small nuclear polymorphisms in both edited and non-edited regions. The high variance within the edited genes in particular may be a fertile source of taxonomic markers to further understand the mitochondrial DNA evolution and RNA editing in *T. cruzi *and other kinetoplastids.

## Results

### Conserved Synteny Among the Three Clades

The three maxicircle coding regions from the three clade taxon representatives Sylvio X10 [GenBank:FJ203996], CL Brener [GenBank:DQ343645] and Esmeraldo [GenBank:DQ343646] were syntenic beginning with the 12S rRNA gene and ending with the ND5 gene (Table 1). Comparing the coding region sequence lengths beginning at the 12S rRNA and ending near the 3' end of ND5 at the equivalent nucleotide sequence for the three maxicircles, Sylvio X10 has 15,185 bp, CL Brener has 15,167 bp, and Esmeraldo has the shortest coding region of 14,935 bp. Only DTU TcII strain Esmeraldo shows a 236-nt deletion at the 5' end of the CR4 and ND4 genes, although this deletion is not characteristic of the DTU [23]; otherwise, the lengths of the coding regions were comparable for individual genes among the three strains. The Sylvio X10 ND5 gene is incomplete due to the absence of conserved sites for oligonucleotide placement within the adjacent maxicircle divergent region, and ~1600 nt of the anticipated ~1770 nt were sequenced.

**Table 1:** Genes in the Sylvio X10, CL Brener and Esmeraldo maxicircle consensus sequences

			position (5'-3')			length	
Gene	Editing	Sylvio	CL Brener	Esmeraldo	Sylvio	CL Brener	Esmeraldo
					CLADE A	CLADE B	CLADE C

12S rRNA	-	1-1159	1-1161	1-1160	1159	1161	1160
9S rRNA	-	1203-1812	1200-1808	1197-1804	610	608	607
ND8	extensive	1856-2135	1853-2131	1957-2127	280	279	271
ND9	extensive	2198-2547	2195-2532	2192-2545	350	338	354
MURF5	none	2541-2801	2526-2789	2522-2788	261	264	267
ND7	extensive	2877-3623	2857-3611	2862-3617	747	755	756
COIII	extensive	3697-4116	3678-4100	2685-4109	420	424	425
Cyb	5' end	4189-5269	4175-5254	4167-5246	1081	1080	1080
ATPase 6	extensive	5310-5639	5292-5627	5288-5622	330	336	335
MURF1	none	5696-7036	5675-7015	5681-7029	1341	1340	1347
CR3*	extensive	7023-7144	7002-7120	7016-7136	~122	~119	~121
NDI	none	7138-8079	7116-8057	7132-8073	942	942	942
COII+gRNA	internal	8093-8737	8071-8715	8088-8733	645	645	646
MURF2	5' end	8748-9800	8725-9780	8750-9794	1053	1056	1045
COI	none	9791-11440	9771-11420	9785-11434	1650	1650	1650
CR4	extensive	11492-11700	11471-11677	11487-11658	209	207	172
MURF2 gRNA	-	11764-11804	11744-11784	-	41	41	-
ND4	none	11802-13114	11782-13095	11659-12872	1313	1314	1214
ND3	extensive	13108-13300	13087-13279	12864-13051	193	193	188
RPS12	extensive	13377-13564	13357-13547	13129-13315	188	191	187
ND5	none	13586-15185**	13568-15337	13335-15105	1600+	1770	1771

Gene positions are given relative to the start of the 12S rRNA

*CR3 5' and 3' end positions are uncertain

**partial sequence

A major revision is included in the annotation of the MURF5 gene for the CL Brener and Esmeraldo strain maxicircles. Whereas MURF5 is described as 5' edited and 147-148 nt in length, the corrected identification is for a non-edited gene of 261-267 nt, depending on the strain. The gene sequence is provided in Additional file 1, Figure S1, along with the predicted translation in alignment including the *T. brucei *predicted protein. MURF5 was notable for the relatively high number of insertion/deletion (indel) mutations throughout the gene, disrupting the open reading frames at the carboxyl-terminus in CL Brener and Esmeraldo.

### Evolutionary Relationship Between the Clade Coding Regions

**Figure 1:**
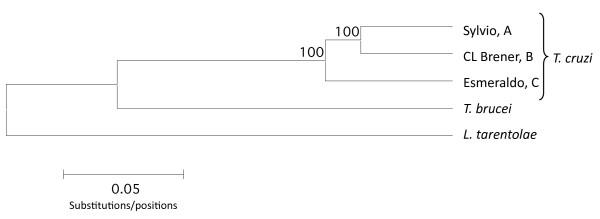
**Phylogenetic relationship among the three clade representatives**. A Neighbor Joining tree shows 100% bootstrap for clade A and B and for clade A and B. The bootstrap and NJ tree were calculated using MEGA 4.0 default parameters and the coding region of the maxicircle from 12S to partial ND5 for each strain. *T. brucei *and *L. tarentolae *were used as outgroups. Sylvio X10 is a representative of clade A; CL Brener is a representative of clade B; Esmeraldo is a representative of clade C.

Phylogenetic analysis was performed using the three *T. cruzi *clades and the *T. brucei *and *L. tarentolae *maxicircles spanning the 12S rRNA gene through the partial ND5 gene from Sylvio X10 (Figure 1). Clades A and B formed a monophyletic group to the exclusion of lineage C in a Neighbor Joining (NJ) tree rooted by *L. tarentolae*. The A and B clades are supported by 100% of the bootstrap replicates, offering significant statistical support for this branching order among clades A, B and C. The tree topology supports previous phylogenetic analyses using either COII-NDI or Cyb genes [18,19].

The relationship between clades A and B validates the model that a DTU TcI strain contributed to the genetic make-up of the heterozygous hybrid DTUs TcV and TcVI. In our genealogy TcI is a 'grandparent' of the heterozygous hybrids, and the maxicircle genome was passed down through a homozygous hybrid TcIII strain [23].

### Maxicircle-encoded gRNA in Sylvio complements CL Brener

**Figure 2:**
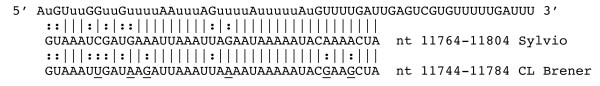
**Maxicircle-encoded Sylvio X10 gRNA for MURF2 RNA editing**. Alignment of the MURF2 gRNA from Sylvio X10 maxicircle aligned with the predicted editing pattern for the 5' end of the MURF2 transcript. Standard Watson-Crick base pairing is shown by '|' and G-U wobble base pairing is represented by ':'. The cognate CL Brener maxicircle-encoded gRNA is shown below. The transitions between the two gRNAs are underlined in CL Brener.

A predicted gRNA gene was found in the Sylvio X10 maxicircle at positions 11764-11804 (Figure 2). An equivalent gRNA from the CL Brener maxicircle is located between the genes for CR4 and ND4 at positions 11744-11784 in the intergenic region, containing the information to direct the RNA editing of the MURF2 gene [24]. The Sylvio X10 maxicircle gRNA overlaps the start codon of the ND4 gene; the post-transcriptionally added poly(U) tail could provide the base pairing downstream of the gRNA coding region for both.

Along the 41-nt length of the predicted gRNA gene, six transitions differentiate the Sylvio X10 and CL Brener gRNA genes. Due to the permissive nature of the gRNA-mRNA interaction, the two gRNAs impart identical editing information, although the 8-bp anchor region that provides the initial interaction between the partially edited mRNA and the gRNA in CL Brener is less stable due to the presence of two wobble pairs. The Esmeraldo maxicircle MURF2 gRNA is lost in the 236-nt CR4/ND4 deletion [23]; a second CL Brener maxicircle gRNA was found downstream of the ND5 gene, beyond the region sequenced for Sylvio X10.

### Average Percent Identities Among Clades A, B, and C

The percent identities for the coding and intergenic regions and the non-edited proteins were calculated using the GLOBAL alignment feature in BioEdit 5.0.9 by pairwise comparisons of the three clades (Table 2). For this analysis, each category of sequence was assembled into a single file for each strain to generate an overall percentage of identity. Minor differences were seen at this level among the three clades in the rRNA or protein-coding genes or for the predicted translations of the non-edited genes, although there is a bias toward a closer relationship between clade A and clade B. The combined intergenic regions showed a wider differentiation among the strains, with clades A and B showing the higher levels of identity. Indel corrections were not made for any strain prior to the comparisons, and the absence of an entire intergenic region in Esmeraldo was likewise not compensated in the other strains. Thus, the equivalent comparisons using strains with intact maxicircles may show higher levels of identity among the protein sequences in particular.

**Table 2:** Average percent identities among the three maxicircle clades

Comparison	Non-edited			Edited	Intergenic
	rRNAs	Genes	Proteins	Genes	

Sylvio vs CL Brener (clade A vs clade B)	93%	92%	86%	93%	81%
CL Brener vs Esmeraldo (clade B vs clade C)	92%	89%	86%	90%	73%
Sylvio vs Esmeraldo (clade A vs clade C)	93%	89%	86%	89%	74%

Non-edited genes: Cyb, MURF1, ND1, COII, MURF2, COI, ND4, partial ND5

Proteins: MURF1, ND1, COII, MURF2, COI

Edited genes: ND8, ND7, COIII, ATPase6, CR4, CR5, RPS12

### Single nucleotide indels in two non-edited regions of Sylvio X10

The presence of indels outside of regions anticipated to be edited relative to the CL Brener maxicircle implied that Esmeraldo was degenerate in at least five genes [23]. While the functional implications for mitochondrial biology are unclear, the maxicircle coding region of Esmeraldo is useful for taxonomic purposes. In the Sylvio X10 coding region, two genes showed indels in their nucleotide sequences that created early stop codons.

**Figure 3:**
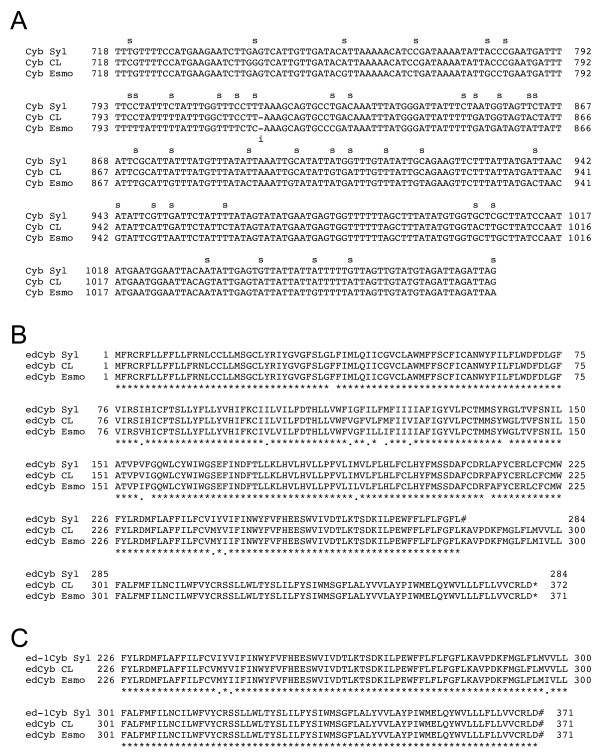
**An insertion in the unedited region of the Sylvio X10 Cyb gene**. A) Portion of the Cyb nucleotide alignment from *T. cruzi *strains Sylvio X10, CL Brener, and Esmeraldo. The Sylvio X10 Cyb gene contains an insertion ('i' under the alignment) at position 817, disrupting the reading frame and resulting in an early stop codon. Positions of single nucleotide polymorphisms (SNPs) are indicated by 's' above the alignment; the distribution of SNPs is compiled in Table 3. B) The predicted Cyb amino acid alignment based on the predicted edited sequence in *T. cruzi *strains Sylvio X10, CL Brener and Esmeraldo. The T insertion present in position 817 causes an early stop codon (#) at position 284. Positions in the alignment with '*' below are identical; '.' indicates a conservative change; a blank represents a non-conservative change or three different amino acids. C) Deletion of Sylvio X10 817 insertion restores the full-length reading frame; the relevant portion of the alignment is shown.

The cytochrome b (Cyb) gene produces a transcript that is 5'-end edited. This 1080-bp gene contained intact reading frames downstream of the presumptive edited region in CL Brener and Esmeraldo, but contained an extra thymidine at nucleotide 818 in the Sylvio X10 sequence (Figure 3A) that resulted in an early stop codon at amino acid 273 (Figure 3B). An intact 360 amino acid ORF is found in CL Brener and Esmeraldo, and deletion of the extra nucleotide in Sylvio X10 restored a conserved ORF through the carboxyl-terminus of the protein (Figure 3C).

**Figure 4:**
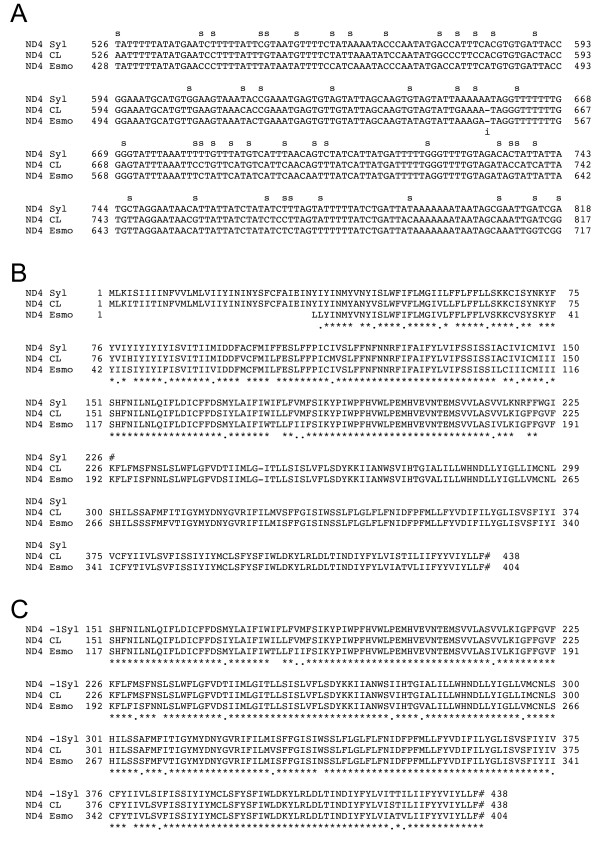
**Insertion in the non-edited ND4 gene in Sylvio X10**. A) Partial representation of the ND4 nucleotide alignment from *T. cruzi *strains Sylvio X10, CL Brener and Esmeraldo. SNPs are indicated by 's' above the alignment; the distribution of SNPs is compiled in Table 3. The Sylvio X10 strain has an insertion ('i' under the alignment) at position 656. B) The ND4 amino acid alignment from the three *T. cruzi *strains; the Esmeraldo sequence is partial due to a 99-nt deletion in the 5' end. An early stop codon (#) is present at position 226 in the Sylvio X10 strain. The available Esmeraldo translation is included for comparison. C) Deletion of the extra A in the Sylvio X10 ND4 gene restores the truncated portion of the protein from aa 226.

The ND4 gene produces transcripts that do not require editing in CL Brener, however in Sylvio X10 this gene contained a single nucleotide insertion at position 656 (Figure 4A) that created an early stop codon at position 226 in the predicted amino acid sequence (Figure 4B). When the nucleotide was deleted manually in the Sylvio X10 sequence, the ND4 transcript can be translated into a complete ORF similar to that of CL Brener (Figure 4C). The Esmeraldo gene contains a major deletion of the 5'-end of the first 99 nts, however the remaining sequence showed conservation of the ND4 coding information.

For both Cyb and ND4, the relatively high levels of sequence identity flanking the Sylvio X10 indels indicated that the overall selective pressure to carry a functional gene is present. The same is true of the compromised gene sequences found in CL Brener and Esmeraldo. Indeed, the indels found in all three genomes point to the poor quality of maxicircle replication, and beg the question of how genome integrity is maintained.

### SNP analysis supports the clade A-clade B relationship

Comparative analysis of maxicircle protein coding genes with little or no RNA editing and their predicted amino acid sequences showed different indels and unique SNPs among the three clades (Table 3). For the alignments used in this analysis, the basic rules that apply to extensively edited genes were used: namely, pyrimidines and purines were scored as functional equivalents. Thus, where an alignment program would introduce an indel where a C and T fell, it was scored as a SNP rather than an indel for this analysis. For the purposes of amino acid comparison, any internal frameshifts were compensated for prior to protein alignment.

**Table 3:** SNPs and indels of non-edited and 5'-edited/minimally edited genes

	SNPs						Amino acid alterations				
Gene	Indels	SNPs	A+B	B+C	A+C	Unique	total	A+B	B+C	A+C	Unique

12S rRNA	18	125	44	37	33	11	-				
9S rRNA	8	46	20	12	14	0	-				
MURF5	17	40	14	12	14	0	27	8	8	4	7
Cyb	1	129	64	36	28	1	15	7	5	3	0
MURF1	10	168	77	47	40	4	29	19	2	7	1
ND1	0	130	43	32	54	1	31	13	10	6	2
COII	0	75	33	18	23	1	17	5	4	8	0
MURF2	20	144	64	35	39	6	-				
COI	0	206	80	62	55	9	8	6	2	0	0
ND4*	2	181	80	44	52	5	38	21	6	8	3
ND5**	7	223	84	79	55	5	55	25	18	7	5

Total	83	1467	603	414	407	43	220	104	55	43	18

*for region beyond Esmeraldo 5' deletion; translation includes correction of frameshift in Sylvio

**through available Sylvio sequence, ~1600 of ~1770 nts; Esmeraldo translation corrected for frameshifts

The prevalence of SNPs varied between 10.8% and 14% among this subset of genes. A total of 155 aa changes resulted from 930 SNPs, revealing that one in six nucleotide mutations resulted in an alteration of amino acid sequence and supporting presence of a selective pressure to maintain the primary protein identity. In contrast to the SNP distribution, the frequency of amino acid alterations varied among the genes within a range of 1.4% for COI to 10.3% for the analyzed portion of the ND5 gene. The presence of indels in specific genes correlated with the nonviable mutations in each strain. The Esmeraldo maxicircle carried the greatest number of indels in this category of gene, with 10 in MURF1, 13 in MURF2, 11 in MURF5, 102 in ND4, and 7 in the partial sequence of ND5. The presence of indels in Cyb and ND4 in Sylvio X10 leave only ND1, COI and COII in functional condition in all three maxicircles among the genes requiring no or minimal editing of their transcripts; the extensively edited genes cannot be judged as easily.

The addition of the Sylvio X10 MURF5 gene to the alignments altered the previous annotations of the CL Brener and Esmeraldo MURF5 genes [23], extending their sequences by over 115 nt each and revealing frameshifts in both at the extreme 3' end of the genes. The resulting ORF is conserved with the *T. brucei *MURF5 gene, lending confidence to the analysis (Additional file 1, Figure S1). The MURF5 gene displayed a high number of deletions for its relatively small size, with a trio of three-nt frame shifts in the front half of the gene that may be acceptable for protein function. In the Sylvio X10 maxicircle, the 3' end of MURF5 overlapped with the first 7 nt of the adjacent ND9 gene, which is transcribed in the same orientation. Thus, the frameshifts in CL Brener and Esmeraldo that throw the termination codon out of frame may result in longer proteins that are still functional. The conservation of primary nucleotide sequence at the 3' end of the genes indicates that the Sylvio X10 frame represents the biologically relevant reference.

At both the cumulative levels of SNPs and of amino acid changes, the linkage between clades A and B was the most pronounced, although not always significant for any particular individual gene. The variability of amino acid replacement for each gene product may reflect the biological tolerance to change as selected by function.

### Extensively edited genes contain abundant thymidine indels

A similar comparative analysis was performed at the nucleotide level for the extensively edited gene set, emphasizing the SNP and indel variability relative to clade (Table 4). Due to the primary sequence variability afforded to these genes by the RNA editing process, standard sequence alignments were not particularly useful, and alignments were performed by eye. For some genes, the actual RNA editing patterns from *T. cruzi *or, in the absence of the *T. cruzi *edited sequence, *T. brucei *were included for comparison.

**Table 4:** SNPs and indels of extensively edited genes

Gene	Indels	A+B	B+C	A+C	SNPs	A+B	B+C	A+C	Unique
ND8	31	19	4	8	6	1	2	2	1
ND9	38	16	16	6	18	13	1	4	
ND7	62	32	11	19	33	14	9	10	
COIII	28	16	7	5	14	5	5	4	
ATPase6	18	8	7	3	17	8	6	3	
CR3	4	3	1	0	4	3	1	0	
CR4*	28	13	11	4	18	8	3	7	
ND3	21	14	4	3	18	8	3	7	
RPS12	11	5	2	4	2	1	1	0	

Total	241	126	63	52	130	61	31	37	1

*beginning after the Esmeraldo deletion

**Figure 5:**
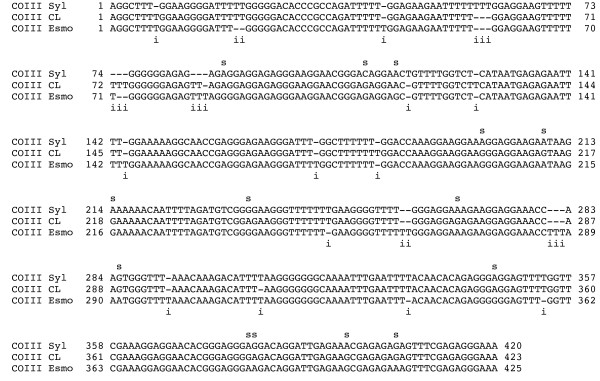
**Abundant indels in extensively edited genes**. The COIII nucleotide sequence alignment using Sylvio X10, CL Brener and Esmeraldo is shown as an example. SNPs are indicated by 's' above the alignment and indels by 'i' below the alignment. In this particular case, indels outnumber SNPs 2:1. The distributions of SNPs and indels for COIII and other extensively edited genes are compiled in Table 4.

The COIII gene illustrated the dramatic effect of RNA editing on the primary sequences among the strains (Figure 5). Although the SNP pattern within these genes were not strong differentiators, the indel patterns were revealing in their associations. A comparison of indels was performed for the COIII gene, revealing 16 shared indels between clades A and B, eight between clades B and C, and five for clades A and C. Due to the unique mechanism of the uridine insertion/deletion RNA editing process, indels involving thymidine residues would be tolerated with a wide degree of latitude. The association of the indel patterns with particular clades cannot be assessed without a broad survey of *T. cruzi *strains, and the permissive nature of the process may prove too accommodating to provide useful taxonomic information. Given the abundance of indels detected among the three clade representatives relative to the size of their respective coding regions, candidate genes for broader indel analysis emerged, including COIII, ND7, ND8, and ND9.

Taken individually each gene showed a wide range of associations among the three clades when considering SNP variability, however the sum total of these differences revealed the bias toward the clade A/B relationship. The number of indels surpassed the SNPs substantially, making them provocative markers for these genes.

**Figure 6:**
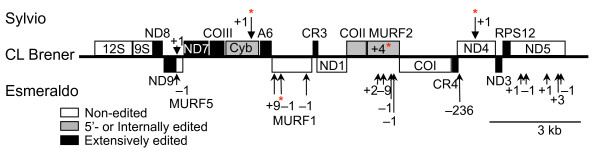
***Trypanosoma cruzi *maxicircle indels in non-edited regions**. Linear representation of the mitochondrial maxicircle for the *T. cruzi *strains Sylvio X10, CL Brener and Esmeraldo with labeled indels and genes. Strain-specific stop codons resulting from indels are marked with a red asterisk (*).

Non-edited genes have higher evolutionary pressure to remain intact when indels cannot be corrected by RNA editing. The indels in areas typically free of RNA editing are summarized for the three clades (Figure 6); this schematic differs from a previous incarnation [23] in that the Sylvio X10 indels have been added, along with several additional indels for Esmeraldo, and genes judged not to be edited in *T. brucei *and *L. tarentolae *despite the absence of canonical start codons are marked accordingly.

## Discussion

The majority of the coding region for *T. cruzi *strain Sylvio X10, a DTU TcI/mitochondrial clade A representative, was sequenced to complete the maxicircle trilogy first defined by Machado and Ayala (2001). The close association between the Sylvio X10 and CL Brener coding regions supports a model in which an ancestor strain of DTU TcI provided the maxicircle to the progeny of a TcI-TcII hybridization event, resulting in the generation of DTUs TcIII and TcIV [23]; a subsequent 'back-cross' hybridization between TcII and TcIII strains resulted in the TcV and TcVI strains that carry the maxicircle from their TcIII/clade B ancestor [7]. The percent identity of clades A and B is the highest across comparisons of rRNA sequences, edited and non-edited genes, amino acid and intergenic regions when compared to the percent identity of clades B and C. The pedigree of the maxicircles provides strong evidence of the parental contribution of DTU TcI in the first fusion event in the genesis of the extant *T. cruzi *population structure. In order to generate useful markers for taxonomic purposes, additional representatives of all clades must be integrated into the analysis; with the complete genome sequencing of other key *T. cruzi *strains in process, the usefulness of the maxicircle will be clarified in the coming years. To better understand the genesis of the extant clades, maxicircles from DTU TcIII are of particular interest as the kDNA donor to the nuclear heterogeneous hybrid lines in DTUs TcV and TcVI.

Perhaps the most striking finding in the study of *T. cruzi *maxicircles is that genes producing transcripts that are not anticipated to be RNA edited in all three strains are compromised in terms of sequence content. The *T. cruzi *clade representatives have the same gene order in the coding region with different number and location of indels and SNPs. Early stop codons are created by indels in the Cyb and ND4 genes of Sylvio X10, in the MURF1 and ND5 genes of Esmeraldo, and in the MURF2 gene of CL Brener; in addition, the Esmeraldo maxicircle has a 236-bp deletion affecting the opposing CR4 and ND4 genes, including the deletion of a gRNA in the intergenic region, and four individual indels in the MURF2 gene. The 5'-edited gene Cyb and the non-edited ND4 found in Sylvio X10 have indels that result in early termination codons that, when adjusted manually, result in conserved ORFs, a general characteristic of all the non-edited region indels. The absence of drift in the sequences downstream of the indels suggests that they are relatively recent. The commonality of affected genes, MURF2 in CL Brener and Esmeraldo and ND4 in Esmeraldo and Sylvio X10, may reflect a hierarchy of tolerable genes for functional loss. Given the ability of the RNA editing mechanism to overcome frameshifts, transcripts from all of the compromised genes must be sequenced. With only two non-edited genes unscathed in any of the three genomes, specifically COI and ND1, the functional necessity of these genes versus their fortuitous exclusion from indel mutation will be clarified with the sequencing of additional maxicircles. The mechanism for the maintenance of these extra-nuclear sequences thus becomes an interesting challenge for the organism. Intriguing suggestions of mitochondrial fusion in the hybridization process may provide the answer [10], as maxicircles could share content information across a heterogeneous population and a form of intercellular copy correction; the occurrence of the fusion event within strains would have to be frequent, whereas a combining of two disparate strains, such as would be required to produce one of the genetic hybrid lines, would be exceedingly rare.

The extensively-edited genes from the Sylvio X10, CL Brener and Esmeraldo strains show that clades A and B share the most SNPs, followed by clades B and C have the second highest and clades A and C having the fewest shared SNPs. Although the gene sequence may vary in the number of thymidines, RNA editing will insert or delete the correct number of uridines to create ORFs that can be translated to produce a functional protein. Each strain has its unique number of indels and SNPs, and comparison of Sylvio X10 and CL Brener shows their close association in comparison to CL Brener and Esmeraldo by this criterion as well.

Phylogenetic analysis of the *T. cruzi *population through the use of microsatellite data has given rise to a different postulated inheritance of the maxicircle genomes, in which clade B (TcIII) is the mitochondrial donor of hybrid DTUs TcV/VI [19]. The maxicircle genealogy shows clade A (TcI) as the mitochondrial donor of hybrid DTUs TcV/VI [23]. Maxicircle coding region comparison of the three clade representatives shows that CL Brener, a DTU TcVI, is associated with the clade A Sylvio X10 strain rather than the clade C Esmeraldo strain. Therefore, the maxicircle was donated from DTU TcIII, an ancient hybrid that inherited its maxicircle from TcI [23].

With the maxicircle sequence available for clade B and C representatives and the majority of the coding region sequenced for clade A representative now complete, further phylogenetic analysis can better define the *T. cruzi *population structure by comparing the editing patterns across the three strains, or sequencing additional maxicircles to see if the similar SNP and indel patterns are followed within clades. The three-clade scheme was based on the two non-edited genes COII and ND1 [18]. Use of the extensively edited genes for comparison across strains from each clade might result in a more refined clade scheme as compared to the phylogenetic relationship observed from maxicircle non-edited genes, and provide further insight into the *T. cruzi *population structure. Alternatively, edited gene indels may behave as microsatellites and prove useful for recent changes in population structure, but be unhelpful for larger analyses.

The existence of complex I involved in electron transfer from NADH to coenzyme Q in trypanosomes has been debated due to the viability of trypanosomes with or without a functional complex I in varying environments and strains [25]. A viable strain without a functional complex I is the UC strain *L. tarentolae *[28]. Some *Trypanosoma *strains can survive with a partial maxicircle, or even with complete loss of kDNA. *Trypanosoma brucei *relatives such as *T. equiperdum *and *T. evansii *are examples that showed gradual reduction or loss of kinetoplastid DNA [29]. *T. equiperdum *has a partial kDNA and *T. evansi *no longer has kDNA, and survive only in the bloodstream stage dependant on glycolysis and down-regulation of the mitochondria [30]. The ability of *T. brucei *strains to find a biological niche for parasitism that frees them from the insect life stages demonstrates the versatility of the kinetoplastids, and may apply equally to *T. cruzi*.

Analysis of the Sylvio X10 maxicircle points to a DTU TcI strain as the mitochondrial donor to hybrid DTUs TcIII/IV, and strengthens our understanding of the clade structure and evolution of *T. cruzi*. With sequencing becoming ever more affordable and accurate [31] we will be able to sequence the mitochondrial maxicircle from representatives of each DTU and re-evaluate the three-clade scheme to redefine or further confirm the population structure as well as uncover markers for strains and clades to better understand Chagas disease.

## Conclusions

With the promise of additional maxicircle genome sequences on the horizon [31], extensive studies will be possible, allowing the quantification of the preliminary observations made with the three clade representatives used here. The use of extensively edited genes as useful taxonomic markers provides a leap beyond the standard mitochondrial DNA, long favored due to its rapid accumulation of mutations. The flexibility afforded by the uridine addition and deletion process may prove to be too high for broad phylogenetic conclusions. The unusual genetic exchange mechanism employed by *T. cruzi *must also be taken into account, as the frequency of this event and its participants are not yet clear. Indeed, the relative lack of diversification within mitochondrial clade B, encompassing both the nuclear homozygous and heterozygous hybrid strains, is surprising, and remains to be examined within the clade.

## Methods

### PCR Amplification

The nucleotide sequence of the maxicircle coding region [GenBank:FJ203996] was obtained by PCR amplification of Sylvio X10 maxicircle using different amplification primers and PCR conditions. The primers pairs along with their sequence, binding site, annealing temperature, MgCl_2 _concentration, region amplified and product size can be found in Table 5. PCR conditions to amplify the maxicircle regions were: 3 min initial denaturation step at 93°C, 30-sec subsequent denaturation at 93°C, primer annealing for 30 sec at temperatures mentioned above, primer extension at 72°C for variable times depending on amplification length, for a total of 30 cycles. Total volume of reaction was 50 μl with *T. cruzi *Sylvio X10 genomic DNA at 0.93 μg, 0.2 mM dNTPs, 0.2 μM forward primer, 0.2 μM reverse primer, 10X standard CLP *Taq *reaction buffer and 5U of NEB standard *Taq *DNA polymerase. PCR products were purified using QIAquick Gel Extraction Kit from QIAGEN. Approximately 2 kb of the divergent region was sequence to complete the 5' end of the 12S rRNA gene and its analysis was not included in this thesis.

**Table 5:** Primers for PCR amplification

Primer name	Primer sequence (5'-3')	Binding site	T_m_	MgCl_2_	Region amplified	Product
			(°C)	(mM)		(nt)

Tc. Sylvio.cons.Region.For1	TTGTAAAAACCTTATCAGCAAAGAAA	Palindrome	46	3.5	Palindrome-partial 12S	~2,000
Tc. Maxi.12S.rRNA.Rev	TTACTTGGTACATATATAACAACTG	12S				
Tc.CLBmaxi12s.For2	GCACAGTTGTTATATATGTAC	12S	50	2.5	partial 12S- partial 9S	1,528
Tc.Sylvio.Maxi9s.2R	GATTACTGCACGTTATTTTTATT	9S				
Tc.9s.For.unedited	GCCCACCAATTTTTATAATAA	9S	38	2.5	partial 9S-partial ND7	1,576
Tc.Maxi.ND8-Murf5.UTR.unedited.Rev	ATCCTTCGAACATCCCTCCT	ND7				
Tc.ND7.For.unedited	CGGGAAGGAAGAACAGTT	ND7	37	2.5	partial ND7-partial Cyb	2,161
Tc.Cyb.Rev.CDS.not edited	CTAATCTAATCTACATACAAC	Cyb				
Sylvio.Cyb.For.CDS.not edited	GTTGTATGTAGATTAGATTAG	Cyb	55	2.5	partial Cyb-partial ND1	2,773
Tc.CL Brener.NDI.CDS.Rev1	TTAATCTTATCAGGATTTGTTAGCC	ND1				
Tc.CL Brener.NDI.CDS.For1	GGCTAACAAATCCTGATAAGATTAA	ND1	50	3.5	partial ND1-partial COI	3,419
Tc.Maxi.4.Rev2	TTGCTTAAGTGTTTCCCACAAA	COI				
Tc.Maxi.4.For	TTTGTGGGAAACACTTAAGCAA	COI	40	2.5	partial COI-partial ND5	2,283
Tc.Maxi.ND3-RPS12.Rev.2	TTGATTGTCAAAAACTTATAAAATGCC	ND5				
Tc. maxiRPS12-ND5.unedited.For1	CCAACTTCCCTTCAAACCAA	RPS12	50	2.5	partial RPS12-partial ND5	1,993
Tc.maxi.ND5.unedited.Rev1	TTCAAAATAACATAACAACATCCGTA	ND5				

### Subcloning and Sequencing

Each PCR product was transformed into TOP-10 cells (Invitrogen) and cloned using the TA-cloning kit (Invitrogen). The PCR primers for each respective PCR product were used for bidirectional sequencing through Laragen, and internal sequencing primers were designed when partial sequences were obtained (Table 6).

**Table 6:** Internal primers

Primer name	Primer sequence (5'-3')	Binding site
99904 2-cons-12s F	CACAAGTTGTTATGCATGTAA	conserved region
99905 2-cons-12s R	CTATCACAATTTGTGGGAAAA	conserved region
83251 13-12S-9S F	CGAAAATAAAATTTTAGTAGCATA	9S
6713 B-9S-ND7 4F	GGTTGCCCTCTTGTTGTCAT	ND9
1-42 CO3 CYB (R)	CCTAAACTGAACCCCACTCC	Cyb
1-43 CO3 CYB (R)	CCTAAACTGAACCCCACTCC	Cyb
1-69 CO3CyB (R )	CAAAAGCAAAGTCGCTCAC	Cyb
1-68-CO3CyB (F)	TTGGAGTTGGGTGGAGTTC	ND7
55103 A Murf4-Cr3 R	CGATTATCTCAGAAAGTGCCTTA	ND1
64986 B-MURF4-CR3 3F	GGCAATGGGAATTGTACCTA	MURF1
6611 B-MRF4-CR3 2F	CGGGCAACAACGGTTTTGA	MURF4 (ATPase6)
69245 COII-CO1 Maxi Sylvio F1	ACCAATTTTGTATAACGCAATTATTA	MURF2
894-17F 4-COII-COI F	CGTATGCTTCTTAATATTATATTT	MURF2
894-18 4-COII-COI R	GCTTGATATAATGCTGCATGA	COI
753-5 CO2-CO1 2F	CGGTATCAATTTTTTGATATA	MURF2
54597 CO2 CO1 2R	CGAGCATTATAAATTCTATTAA	COI
55799 CO2-CO1 F	GTAGAGAACCGGGGAGGTGT	COII
57799 CO2-CO1 R	TTGCACCTGTTATGGTTGGAT	COI
1-58-ND4CR4Bfor-M13F_B08	GGAGACTTTTTTACCAAGGG	ND3
1-59-ND4CR4Brev-M13R_CO8	TCCCCCTTCTTCTCCTTCAC	CR4
259-67 RPS12-ND5 F	TGGCTAACCTTTTCATGTTCA	ND5
256-66 RPS12-ND5 R	CCTTGCAATAAAATCCACACAA	ND5

### Construction of the Sylvio X10 Maxicircle

Raw sequence data from chromatograms was analyzed and edited using BioEdit 5.0.9 [32]. The PCR regions amplified were aligned using BioEdit 5.0.9 by overlapping the scaffolds. Annotation of Sylvio X10 maxicircle genes [GenBank:FJ203996] was done manually by comparing the published CL Brener [GenBank:DQ343645] and Esmeraldo [GenBank:DQ343646] maxicircle coding region sequences. BioEdit 5.0.9 was used to calculate percent identity. As only a few RNA editing events have been documented in *T. cruzi*, edited sequences were inferred based on our previous analysis of *T. cruzi *maxicircles or using the cognate editing events in *T. brucei *(see Additional file 1, Figure S1 for the complete analysis).

### Phylogenetic Analysis

The neighbor-joining tree was calculated using the program MEGA 4.0 and its default parameters using LodDet distance. The bootstrap values were calculated using the same method. The NJ tree was constructed using the nucleotide sequences of the coding region beginning at the 12S rRNA gene and ending near the 3' end of the ND5 gene from Sylvio X10, CL Brener and Esmeraldo. We used the same coding region of *L. tarentolae *and *T. brucei *as outgroups.

## Abbreviations

CO: cytochrome oxidase; CR: C-rich region; Cyb: apocytochrome b; DTU: discrete typing unit; gRNA: guide RNA; indels: insertion/deletion mutations; kDNA: kinetoplast DNA; MURF: mitochondrial unidentified reading frame; ND: NADH dehydrogenase; NJ: Neighbor Joining; SNPs: single nucleotide polymorphism.

## Authors' contributions

LIRT carried out the amplification, cloning, sequencing, and bioinformatic analysis of the Sylvio XL-10 maxicircle, and drafted the manuscript. NRS conceived of the project, participated in design and analysis, and drafted the manuscript. Both authors read and approved the final manuscript.

## Authors' Information

Currently LIRT works in Licensing and Technology Acquisitions at Santa Cruz Biotechnology.

## Supplementary Material

Additional file 1 **Figure S1 - Alignments of all maxicircle genes with SNPs and indels indicated for the three strains for which the coding regions are available in their entirety**. The genes are presented in linear order along the length of the maxicircle, beginning with the 12S rRNA gene. SNPs are indicated by 's' above the alignment; indels by 'i' below the alignments; for the ND5 analysis 'v' indicates a transversion SNP. Predicted *T. cruzi *edited sequences highlit in green differ from the new predictions for ND8, which are based on *T. brucei *editing events. The MURF5 annotation and alignment differs from our previous report [23], and shows the predicted amino acid alignment including the *T. brucei *sequence. Click here for file

## References

[B1] Teixeira AR Arganaraz ER Freitas LH Jr Lacava ZG Santana JM Luna H Possible integration of *Trypanosoma cruzi *kDNA minicircles into the host cell genome by infection Mutat Res 1994 305 197 209 7510031 10.1016/0027-5107(94)90240-2

[B2] Campbell DA Westenberger SJ Sturm NR The determinants of Chagas Disease: Connecting parasite and host genetics Curr Mol Med 2004 4 549 562 10.2174/1566524043360249 15357207

[B3] Lukeš J Guilbride DL Votypka J Zikova A Benne R Englund PT Kinetoplast DNA network: evolution of an improbable structure Eukaryot Cell 2002 1 495 502 12455998 10.1128/EC.1.4.495-502.2002 PMC117999

[B4] Simpson L Sbicego S Aphasizhev R Uridine insertion/deletion RNA editing in trypanosome mitochondria: a complex business RNA 2003 9 265 276 10.1261/rna.2178403 12591999 PMC1370392

[B5] Gaunt MW Yeo M Frame IA Stothard JR Carrasco HJ Taylor MC Solis Mena S Veazy P Miles GAJ Acosta N Mechanism of genetic exchange in American trypanosomes Nature 2003 421 936 939 10.1038/nature01438 12606999

[B6] Westenberger SJ Barnabé C Campbell DA Sturm NR Two hybridization events define the population structure of *Trypanosoma cruzi* Genetics 2005 171 527 543 10.1534/genetics.104.038745 15998728 PMC1456769

[B7] Sturm NR Campbell DA Alternative lifestyles: The population structure of *Trypanosoma cruzi* Acta Trop 2010 115 35 43 10.1016/j.actatropica.2009.08.018 19695212

[B8] Sturm NR Teixeira A, Vinaud MC, Castro AM *Trypanosoma cruzi *mitochondrial DNA and the parasite lifecycle Emerging Chagas Disease 2009 Bentham Science Publishers 63 69

[B9] Campbell DA Sturm NR Teixeira A, Vinaud MC, Castro AM *Trypanosoma cruzi *nuclear DNA and its correlation with the parasite life-cycle Emerging Chagas Disease 2009 Bentham Science Publishers 70 82

[B10] Carranza JC Valadares HMS D'Ávila DA Baptista RP Moreno M Galvão LMC Chiari E Sturm NR Gontijo ED Macedo AM Zingales B *Trypanosoma cruzi *maxicircle heterogeneity in Chagas disease patients from Brazil Int J Parasitol 2009 39 963 973 10.1016/j.ijpara.2009.01.009 19504756

[B11] Brisse S Barnabé C Tibayrenc M Identification of six *Trypanosoma cruzi *phylogenetic lineages by random amplified polymorphic DNA and multilocus enzyme electrophoresis Int J Parasitol 2000 30 34 44 10.1016/S0020-7519(99)00168-X 10675742

[B12] Brisse S Dujardin J-C Tibayrenc M Identification of six *Trypanosoma cruzi *lineages by sequence-characterised amplified region markers Mol Biochem Parasitol 2000 111 95 105 10.1016/S0166-6851(00)00302-9 11087920

[B13] Zingales B Andrade SG Briones MR Campbell DA Chiari E Fernandes O Guhl F Lages-Silva E Macedo AM Machado CR A new consensus for *Trypanosoma cruzi *intraspecific nomenclature: second revision meeting recommends TcI to TcVI Mem Inst Oswaldo Cruz 2009 104 1051 1054 10.1590/S0074-02762009000700021 20027478

[B14] Sim˜es-Barbosa A Argañaraz ER Barros AM Rosa Ade C Alves NP Louvandini P D'Souza-Ault MR Nitz N Sturm NR Nascimento RJ Teixeira AR Hitchhiking *Trypanosoma cruzi *minicircle DNA affects gene expression in human host cells via LINE-1 retrotransposon Mem Inst Oswaldo Cruz 2006 101 833 843 17293976 10.1590/s0074-02762006000800003

[B15] Hecht MM Nitz N Araujo PF Sousa AO Rosa Ade C Gomes DA Leonardecz E Teixeira A R L Inheritance of DNA transferred from American trypanosomes to human hosts PLoS One 2010 5 e9181 10.1371/journal.pone.0009181 20169193 PMC2820539

[B16] Baptista CS Vencio RZ Abdala S Carranza JC Westenberger SJ Silva MN Pereira CA Galvao LM Gontijo ED Chiari E Differential transcription profiles in *Trypanosoma cruzi *associated with clinical forms of Chagas disease: Maxicircle NADH dehydrogenase subunit 7 gene truncation in asymptomatic patient isolates Mol Biochem Parasitol 2006 150 236 248 10.1016/j.molbiopara.2006.08.008 16996148

[B17] Morel C Chiari E Camargo EP Mattei DM Romanha AJ Simpson L Strains and clones of *Trypanosoma cruzi *can be characterized by pattern of restriction endonuclease products of kinetoplast DNA minicircles Proc Natl Acad Sci USA 1980 77 6810 6814 10.1073/pnas.77.11.6810 6256762 PMC350379

[B18] Machado CA Ayala FJ Nucleotide sequences provide evidence of genetic exchange among distantly related lineages of *Trypanosoma cruzi* Proc Natl Acad Sci USA 2001 98 7396 7401 10.1073/pnas.121187198 11416213 PMC34680

[B19] de Freitas JM Augusto-Pinto L Pimenta JR Bastos-Rodrigues L Goncalves VF Teixeira SM Chiari E Junqueira AC Fernandes O Macedo AM Ancestral Genomes, Sex, and the Population Structure of *Trypanosoma cruzi* PLoS Pathog 2006 2 e24 10.1371/journal.ppat.0020024 16609729 PMC1434789

[B20] Junqueira AC Degrave W Brandão A Minicircle organization and diversity in *Trypanosoma cruzi *populations Trends Parasitol 2005 21 270 272 10.1016/j.pt.2005.04.001 15922247

[B21] Brisse S Henriksson J Barnabé C Douzery EJP Berkvens D De Carvalho MRC Buck GA Dujardin J-C Tibayrenc M Evidence for genetic exchange and hybridization in *Trypanosoma cruzi *based on nucleotide sequences and molecular karyotype Infect Genet Evol 2003 2 173 183 10.1016/S1567-1348(02)00097-7 12797979

[B22] El-Sayed NM Myler PJ Bartholomeu DC Nilsson D Aggarwal G Tran AN Ghedin E Worthey EA Delcher AL Blandin G The genome sequence of *Trypanosoma cruzi*, etiologic agent of Chagas disease Science 2005 309 409 415 10.1126/science.1112631 16020725

[B23] Westenberger SJ Cerqueira GC El-Sayed NM Zingales B Campbell DA Sturm NR *Trypanosoma cruzi *mitochondrial maxicircles display species- and strain-specific variation and possess a conserved element in the non-coding region BMC Genomics 2006 7 60 10.1186/1471-2164-7-60 16553959 PMC1559615

[B24] Thomas S Martinez LL Westenberger SJ Sturm NR A population study of the minicircles in *Trypanosoma cruzi*: predicting guide RNAs in the absence of empirical RNA editing BMC Genomics 2007 8 133 10.1186/1471-2164-8-133 17524149 PMC1892023

[B25] Opperdoes FR Michels PA Complex I of Trypanosomatidae: does it exist? Trends Parasitol 2008 24 310 317 10.1016/j.pt.2008.03.013 18534909

[B26] Carranza JC Kowaltowski AJ Mendonça MA de Oliveira TC Gadelha FR Zingales B Mitochondrial bioenergetics and redox state are unaltered in *Trypanosoma cruzi *isolates with compromised mitochondrial complex I subunit genes J Bioenerg Biomembr 2009 41 299 308 10.1007/s10863-009-9228-4 19618257

[B27] Verner Z Cermáková P Skodová I Kriegová E Horváth A Lukeš J Complex I (NADH:ubiquinone oxidoreductase) is active in but non-essential for procyclic *Trypanosoma brucei* Mol Biochem Parasitol 2011 175 196 200 10.1016/j.molbiopara.2010.11.003 21074578

[B28] Thiemann OH Maslov DA Simpson L Disruption of RNA editing in *Leishmania tarentolae *by the loss of minicircle-encoded guide RNA genes EMBO J 1994 13 5689 5700 7988566 10.1002/j.1460-2075.1994.tb06907.x PMC395534

[B29] Lai DH Hashimi H Lun ZR Ayala FJ Lukes J Adaptations of *Trypanosoma brucei *to gradual loss of kinetoplast DNA: *Trypanosoma equiperdum *and *Trypanosoma evansi *are petite mutants of T. brucei Proc Natl Acad Sci USA 2008 105 1999 2004 10.1073/pnas.0711799105 18245376 PMC2538871

[B30] Besteiro S Barrett MP Rivière L Bringaud F Energy generation in insect stages of *Trypanosoma brucei*: metabolism in flux Trends Parasitol 2005 21 185 191 10.1016/j.pt.2005.02.008 15780841

[B31] Sturm NR Martinez LL Thomas S Kinetoplastid genomics: the thin end of the wedge Infect Genet Evol 2008 8 901 906 10.1016/j.meegid.2008.07.001 18675383 PMC2676795

[B32] Hall TA BioEdit: A user-friendly biological sequence alignment editor and analysis program for Windows 95/98/NT Nucleic Acids Symp Ser 1999 41 95 98

