# Supplementary material for: The Trypanosoma cruzi Sylvio X10 strain maxicircle sequence: the third musketeer
Source: BMC Genomics. 2011 Jan 24;12:58. doi: 10.1186/1471-2164-12-58 (PMC3040149; doi:10.1186/1471-2164-12-58)
Supplement: Additional file 1 — Figure S1 - Alignments of all maxicircle genes with SNPs and indels indicated for the three strains for which the coding regions are available in their entirety. The genes are presented in linear order along the length of the maxicircle, beginning with the 12S rRNA gene. SNPs are indicated by 's' above the alignment; indels by 'i' below the alignments; for the ND5 analysis 'v' indicates a transversion SNP. Predicted T. cruzi edited sequences highlit in green differ from the new predictions for ND8, which are based on T. brucei editing events. The MURF5 annotation and alignment differs from our previous report [23], and shows the predicted amino acid alignment including the T. brucei sequence. [file 1471-2164-12-58-S1.PDF]

12S rRNA

```
                 ss       s      s s             sss   s            sss     ss     ss
Syl 12S      1 ATGTCATCAAGTTATTTTGAATTTATAATAATGATTC-TTGAT-TTTTTTATGTAAGGTATACAAATTTTATATA   73
CL 12S       1 ATATCATCAAGTTCTTTTGAATTTATAATAATGACCAATTAAT-TTTTTTATGTGAG-TATTTAAATTCTATATA   73
Esmo 12S     1 ATTGCATCAAGAT-TTTTAAGTTTATAATAATGATTAATTAATATTTTTTATGCAGG-TATTTAAATTCCATATA   73
                            i                       i     i             i

                s             s        s   s sss
Syl 12S     74 GTATTATTATTATTTATTTATTTATTTATTTAGTGTGTTATATTTAAGTATTAAATTTGTTGTTTTATATTTAGT  148
CL 12S      74 GTATTATTATTATTTGTTTATTTAATTAGTATATGTGTTATATTTAAGTATTAAATTTGTTGTTTTATATTTAGT  148
Esmo 12S    74 GCATTATTATTATTTGTTTATTTAATTAATATGTGTGTTATATTTAAGTATTAAATTTGTTGTTTTATATTTAGT  148

                ss  s   ss s   s  s        ss  s                       s  s s      ss
Syl 12S    149 TTTATATATTGTTATTGA-TATAAGGAAGGTATAATTTTAAAATTTACTGAACTGTGATGTATAGTTTGTTATTT  222
CL 12S     149 TTTATTTATCATCATTAACCATAAGGAAAATATAATTTTAAAATTTACTGAACTGTGATATGTAGTTTATTATTT  223
Esmo 12S   149 TAAATATATTATTATTAACTATAAGGAAAATACAATTTTAAAATTTACTGAACTGTAATATATAGTTTACTATTT  223
                                 i

                                                ss                            s         ss
Syl 12S    223 TTTGTTTAATGTTTAAATATTTAACTATTGAAGAC-ACAGTTGTTATATATGTACCAAGTAAAAATAGTAAGAAC  296
CL 12S     224 TTTGTTTAATGTTTAAATATTTAACTATTGAAGGC-ACAGTTGTTATATATGTACCAAGTAAAAATAGTAAGAGT  297
Esmo 12S   224 TTTGTTTAATGTTTAAATATTTAACTATTGAAGGTTACAGTTGTTATATATGTACCAAGTAAAGATAGTAAGAGT  298
                                                  i

                   s    s s                       ss sss s   s           s s             s
Syl 12S    297 ATTTTATTTGAATTAATAAATAATTATTTAA--TTTATTGGAAAATAAATATTATGAAGACTTTAAAAATTAATA  369
CL 12S     298 ATTTCATTTAAATTAATAAATAATTATTTAAAATTATTGGAATAATGAATATTATGAAAACTTTAAAAATTAATA  372
Esmo 12S   299 ATTTTATTTAATTTAATAAATAATTATTTAA--TTTTTAAAATAATAAATATTATGAAAATTTTAAAAATTAATC  371
                                              ii

                             s   ss ss sssssss   s  s    s    s  s                 s  s  s
Syl 12S    370 TTTTTAACTGATAATAAATCATAAGCATCACATTTATTTGAACATTAAAGCTACATATTTAATTTGTAATTTATG  444
CL 12S     373 TTTTTAACTGATAACAAAGTATAAGTTATTCATTCATCTGAATATTAGAGATACATATTTAATTTGTAATTTATG  447
Esmo 12S   372 TTTTTAACTGATAATAAAAAAATATAATTATATTTATTTGAATATTAAAGATACATATTTAATTTGTACTTCATT  446

                      ss s s ssssss s      s       s  s s  s    s
Syl 12S    445 TACATTACTACAATGCCAAAAATCAGTAATAAAATAAAAGAG-TCAATATA-AAAAATTAATATTTAATATTTGT  517
CL 12S     448 TG--TTATTATAC-TATAAAAGTCAGTAATAAAATAGAAAAG-TTAATACAAAAAAATTAATATTTAATATTTGT  518
Esmo 12S   447 AC--ATATAATATATGATTTAATCAGTAGTAAAATAGAAAAAATCAATACAGAAAAATTAATATTTAATATTTGT  519
                 ii         i                            i        i

                s   s      s    s   ss  s   ss              ss
Syl 12S    518 TCAATCAAAAGTAACTTAA--ATTTCAAAGCATAAGTATTATTATTAAAAATATAATTTTAAAAATATAAATATA  590
CL 12S     519 TTAATTAAAAGTGACTTGATATGTTTAAAGCATAAGTATTATTATTAAAAATATAATTTTAAAAATATAAATATA  593
Esmo 12S   520 TTAATCAAAAGTAACTTAATGATTTTAAATTATAAGTATTATTATAGAAAATATAATTTTAAAAATATAAATATA  594
                                  ii

                s                                                                  s
Syl 12S    591 TCGTTAATAAAATTATTGTATTTTAAGTGCGTTTATTAAATGCGTTTGTCTAAGAAATATATTTAAGATTATTCT  665
CL 12S     594 TTGTTAATAAAATTATTGTATTTTAAGTGCGTTTATTAAATGCGTTTGTCTAAGAAATATATTTAAGATTATTCT  668
Esmo 12S   595 TTGTTAATAAAATTATTGTATTTTAAGTGCGTTTATTAAATGCGTTTGTCTAAGAAATATATTTAAGAGTATTCT  669

                                                       s  s       s
Syl 12S    666 TGTATATATATTTATAAATTAATAATTCTAAAAATAAAATAAAGCTTCCTCGAAAATAAAATTTTAGTAGCATAG  740
CL 12S     669 TGTATATATATTTATAAATTAATAATTCTAAAAATAAAATAAAACTTCCTCGAAAATAAAATTTTAGTAGCATAG  743
Esmo 12S   670 TGTATATATATTTATAAATTAATAATTCTAAAAATAAAATGAAACTTCCTCAAAAATAAAATTTTAGTAGCATAG  744

Syl 12S    741 TAATTTGTTAACTAATTATTAAAGTGTTCCACAGAAATTTTTTAAATTAAAACAAAAAAAATAATTTATGAATTA  815
CL 12S     744 TAATTTGTTAACTAATTATTAAAGTGTTCCACAGAAATTTTTTAAATTAAAACAAAAAAAATAATTTATGAATTA  818
Esmo 12S   745 TAATTTGTTAACTAATTATTAAAGTGTTCCACAGAAATTTTTTAAATTAAAACAAAAAAAATAATTTATGAATTA  819

Syl 12S    816 AAATAAAAATTTTAATAAAAAATTAAATAATTAAAATAGGGCAAGTCCTACTCTCCTTTACAAAGAGAACGTTTCA  890
CL 12S     819 AAATAAAAATTTTAATAAAAAATTAAATAATTAAAATAGGGCAAGTCCTACTCTCCTTTACAAAGAGAACGTTG-A  892
Esmo 12S   820 AAATAAAAATTTTAATAAAAAATTAAATAATTAAAATAGGGCAAGTCCTACTCTCCTTTACAAAGAGAACGT---A  891
                                                                      iii
               sss                                                  s
Syl 12S    891 ATAATACGTAATTGTATGTTTGATTGGGGCAATACTATATTTATTTATATAGGCAAAGAACTATAATTATTGAAA  965
CL 12S     893 CATATACGTAATTGTATGTTTGATTGGGGCAATACTATATTTATTTATATAGGGAAAGAACTATAATTATTGAAA  967
Esmo 12S   892 AAAATACGTAATTGTATGTTTGATTGGGGCAATACTATATTTATTTATATAGGTAAAGAACTATAATTATTGAAA  966
```

```
                          s                        s
Syl 12S    966 TAATAAAAGGTTCGAGCAGGTTAACAAGCATTAATACTAAATGTGTTTCATCGTCTACTTATTGCTAAACAAATT 1040
CL 12S     968 TAATAAAAGGTCCGAGCAGGTTAACAAGCATTAATACTAAATGTGTTTCATCGTCTACTTATTGCTAAACAAATT 1042
Esmo 12S   967 TAATAAAAGGTTCGAGCAGGTTAACAAGCATTAATATTAAATGTGTTTCATCGTCTACTTATTGCTAAACAAATT 1041


Syl 12S   1041 GATTGTTCATCAAATTTGCAATTCGTTAGTTGGGTTAAAATCGTTGTAAAGCAGATTTGTTTATATATTTAATTA 1115
CL 12S    1043 GATTGTTCATCAAATTTGCAATTCGTTAGTTGGGTTAAAATCGTTGTAAAGCAGATTTGTTTATATATTTAATTA 1117
Esmo 12S  1042 GATTGTTCATCAAATTTGCAATTCGTTAGTTGGGTTAAAATCGTTGTAAAGCAGATTTGTTTATATATTTAATTA 1116


               s    ssss  ss s      s               s
Syl 12S   1116 ATATAAGGAGTGCATAGTAATATTAGTACGCAAGGATCAATTAT   1159
CL 12S    1118 ATATAGGTAGTTAATAGTAATGTTAGTACGCAAGGATTAATTAT   1161
Esmo 12S  1117 TTATAGTGGGTTTACAGTAATGTTAGTACGCAAGGATCAATTAT   1160
```

9S rRNA

```
                  s                                                          s  s s    s
Syl 9S     1 AAGTCTATTGTTATTATTCATATTAATTTTTTTAAAAGTTTTTAAGTTTTATGCTAGTTTATTTTAAAAT-AATT  74
CL 9S      1 AAGTCCATTGTTATTATTCATATTAATTTTTTTAAAAGTTTTTAAGTTTTATGCTAGTTTATTTTAAGACCAATA  75
Esmo 9S    1 AAGTCTATTGTTATTATTCATATTAATTTTTTTAAAAGTTTTTAAGTTTTATGCTAGTTTATTTCAAAATTAATA  75
                                                                                   i

             s    ss   s                                          s         s
Syl 9S    75 TTATTTGTCATATTTTAGGAATAGATAATAATAATTTATAATTTTGATTAGATGTATTTGTTAATGCTATTTAAT 149
CL 9S     76 ATATTCATCACATTTTAGGAATAGATAATAATAATTTATAATTTTGATTAGATATATTTGTTAGTGCTATTTAAT 150
Esmo 9S   76 A-ATTTGTCATATTTTAGGAATAGATAATAATAATTTATAATTTTGATTAGATGTATTTGTTAGTGCTATTTAAT 149
              i

                           s        s s     s  ss    s         s      s
Syl 9S   150 GGGTGTGGAAATATGTATAAAATTTAATAAAATATTATTACAATAAATTACATTT-ACATATTAGTCAGAAATGG 223
CL 9S    151 GGGTGTGGAAATATTTATAAAATCTAATAAAATATTATTATAATAAATTATATTT-ACATATTAGTCAGAAATGG 224
Esmo 9S  150 GGGTGTGGAAATATTTATAAAATTTGATAAAGTACCATTATAATAAATTACATTTTATATATTAGTCAGAAATGG 224
                                                                    i

                                                                s                 s   s
Syl 9S   224 ATGCCAGCCGTTGCGGTAATTTCTATGCTTTTAAATATTATACATTTATCATAAAA-TATTGTTATTTTTATACT 297
CL 9S    225 ATGCCAGCCGTTGCGGTAATTTCTATGCTTTTAAATATTATACATTTATCATAAAA-TATTGTTACTTTTATACT 298
Esmo 9S  225 ATGCCAGCCGTTGCGGTAATTTCTATGCTTTTAAATATTATACATTTATCACAAAAATATTGTTA-TTTCATATT 298
                                                                     i        i

                s         sss s s          s                                  ss
Syl 9S   298 TTAACAAAAAAAATAAAAGCAACATATCTTTTTTGTTTTTAAACACCTTTTGGTATATGCAAATACGAAATTATA 372
CL 9S    299 TTAGCAAAAAAAATAAAAGAA--ATATCTTATTTGTTTTTAAACACCTTTTGGTATATGCAAATATAAAATTATA 371
Esmo 9S  299 TTAACAAAAAAAAATTAGGAA---TATCTTTTTTGTTTTTAAACACCTTTTGGTATATGCAAATATAAAATTATA 370
                                  iii

                                                        ss     ss         s       s
Syl 9S   373 GTAATTATATATTATATTATATTATATTTATTCATATAATTAACGCATAAAAAGTAATTAAATTTGATACCATGC 447
CL 9S    372 GTAATTATATATTATATTATATTATATTTATTCATATAATTAATACATAAAAAGTAATTAATTTTGATACCATGC 446
Esmo 9S  371 GTAATTATATATTATATTATATTATATTTATTCATATAATTAACACATAAGGAGTAATTAATTTTGATATCATGC 445

                  ss                                 s    ss          s ss
Syl 9S   448 TAAGGCAAAAATATTGAAATTGTAAATGTTATAATCAAAAGTTACTATTTATATAAAATTCTAAATTTTAGATAA 522
CL 9S    447 TAAGGTTAAAATATTGAAATTGTAAATGTTATAATCAAAAATTACCATTTATATAAAATTTTAAATTTTAGATAA 521
Esmo 9S  446 TAAGGTTAAAATATTGAAATTGTAAATGTTATAATCAAAAGTTATTATTTATATAAAGTCTTAAATTTTAGATAA 520

                        s                                                   s
Syl 9S   523 AATAATAAATTCAAAAGGTATTGTTGCCCACCAATTTTTATAATAAAAATAACGTGCAGTAATCAATATATTTAT 597
CL 9S    522 AATAATAAATTCAAAAGGTATTGTTGCCCACCAATTTTTATAATAAAAATAACGTGCAGTAATCAATATATTTAT 596
Esmo 9S  521 AATAATAAATTTAAAAGGTATTGTTGCCCACCAATTTTTATAATAAAAATAACGTGCAGTAATTAATATATTTAT 595

Syl 9S   598 AAAAATATATTTT 610
CL 9S    597 AAAAATATATTTT 609
Esmo 9S  596 AAAAATATATTTT 608
```

ND 8

```
ATTATTTTTTTATTTTTTTATTTTTGTATGTTTTTTTTTGATTTTTTGTTTTTTTTTTTTGTTTGTTTTTATATGTGTTTTGTT      Tb edited
 I  I  F  L  F  F  Y  F  C  M  F  F  F  D  F  L  F  F  F  F  V  C  F  Y  M  C  F  V       Tb aa
AuUAuuuuuuuAuuuuuuUAuuuuuAuAuGuuuuuuuuuGAuuuuuuAuuuuuuuUUUUGuuuGuuuuuAuAuGuGUUUUGuu      Sylvio edited
AuCAuuuuuuUAuuuuuuuAuuuuUAuAuGuuuuuuuuuGAuuuuuuAuuuuuuuUUUUGuuuGuuuuuAuAuGuGuUUUGuu      SW CL edited
  s
A TA       A      TA     A A G         GA      A       TTTTTG   G     A A G GTTTTG       Sylvio DNA
A CA      TA        A    TA A G         GA      A       TTTTTG   G     A A G G TTTG       CL Brener DNA
A GA       A     TCA     ATA G         GA      A        TTTTG   G     A A G GTTTTG       Esmeraldo DNA
          i      ii     i i                            i                     i

TGTTGTGTTACTATTTGTTT  A   CCCATTGAGTTAACCATTGTTAGTTTATTGGTTCGTGGTAA       CCATTTTTTGCGTTTTTA  TTGG
 C  G  V  T  I  C  L       P  I  E  L  T  I  V  S  L  L  V  R  G  N         H  F  L  R  F  Y    W
uGuuGuGuuACCAUUUGuUU  A   CCCAuuGAAuuAACAAuuGuuAGuuuAuuAGUCCGuGGuAA       UCAuuuuuuGCGuUUUUA  UUGA
uGuuGuGuuACCAUUUGuUU  A   CCCAuuGAAuuAACAAuuGuuAGuuuAuuAGUuCCuGGuGA       ACAuuuuuuGCGUUUUuA  UUGG
                                                                                                 s
 G  G G  ACCATTTG TT  A TTCCCA  GAA  AACAA  G  AG  A  AGTCCG GG AATTTTT   CA      GCGTTTT A TTTGA
 G  G G  ACCATTTG TT  A TTCCCA  GAA  AACAA  G  AG  A  AGTCCG GG AATTTTTTTTCA      GCG TTTTA TTTGG
 G  G G  ACCA TTGCTTTTATTTCCCA  GAA  AACAA  G  AG  A  AGTCCG GGCAA        CA      GCG TTTTATTTTGG
            i   i  ii i                                         i  iiiiiii           i     i

TGTGGTTTAGAGCGTTGTATTGCTTGTCGTTTATGTGATTTAATTTGCCCTA     GTTTAGCATTGGATG  TTCGTGTTGGGTG   GAGT
 C  G  L  E  R  C  I  A  C  R  L  C  D  L  I  C  P       S  L  A  L  D  V     R  V  G  W     S
uGuGGuuuGGAACGuuGuAuuGCuuGuCGuuuAuGuGAuuuGAUUUGCCCuA     GuuuAGCAuuGGAuG  UUCGuGuuGGAuG    AAGu
uGuGGuuuAGAACGuuGuAuuGCuuGuCGuuuAuGuGAuuuGGUUuGCCCuA     GuuuAGCAuuGGAuG  UUCGuGuuGGAuAuuuAAGu
        s                                 s                                           s
 G GG   GGAACG  G A  GC  G CG  A G GA  GATTTGCCC ATTTTTG  AGCA  GGA GTTTTCG G  GGA G  AAG
 G GG   AGAACG  G A  GC  G CG  A G GA  GGTT GCCC A TTTTG  AGCA  GGA GTTTTCG G  GGA A  AAG
 G GG   GGAACG  G A  GC  G CG  A G GA  GG   GCCC A TTTTG  AGCA  GGA GTTTTCG G  GGA G  AAG
                                          iii      i

TTTGGTGGTCATCGTTTTGCGGATTG   ATTTACATTGAGTTATCGTC     GTTGTATTTATTGTGGTTTTTGTATGCATGTTTGCCCG
 F  G  G  H  R  F  A  D  W     F  T  L  S  Y  R       R  C  I  Y  C  G  F  C  M  H  V  C  P
uuuGGuGGCCACCGuuuuGCCGAuuG   AuuuACAuuAAGuuACCGCC     GuuGuAuuuAuuGuGGuuuuuGuAuGCAuGuuuGCCCG
uuuGGuGGCCACCGuuuuGCCGAuuGUUUA   ACAuuAAGuuACCGCC     GuuGuAuuuAuuGuGGuuuuuGuAuGCAuGuuuGCCCG

   GG GGCCACCG   GCCGA  GTTTA   ACA  AAG  ACCGCCTTTT G  G A   A  G GG    G A GCA G   GCCCG
   GG GGCCACCG   GCCGA  GTTTA   ACA  AAG  ACCGCCTTTTTG  G A   A  G GG    G A GCA G   GCCCG
   GG GG CACCG   GCCGA  GTT A   ACA  AAG  ACCGCCTTTTTG  G A   A  G GG    G A GCA G   GCCCG
        i                   i                        i

ACAGA    TGCCATTACGCATTCATTGTTTGTTATGTGTTTTTGTTGTTTAGCCA TGTATTTATTG    GCGC     C      CAA  G
 T  D      A  I  T  H  S  L  F  V  M  C  F  C  C  L  A   M  Y  L  L      A       P       K
ACGGA    UGCCAuuACACAUUCAuuGuuuGuuAuGuGuuuuuGuuGuuuAGCCA UGuAuuuAuuG    GCAC     C      CAA  G
ACGGA    UGCCAuuACACAUUCAuuGuuuGuuAuGuGuuuuuGuuGuuuAGCCA UGuAuuuAuuG    GCAC     C      CAA  G
  s
ACGGA TTTTGCCA  ACACATTCA  G   G  A G G     G  G   AGCCATTG A   A  GTTTTGCACTTTTCTTTTTTCAATTG
ACGGATTTTTGCCA  ACACATTCA  G   G  A G G     G  G   AGCCATTG A   A  GTTTTGCACTTTTC TTTTTCAATTG
ACAGA TTTTGCCA  ACACATTCA  G   G  A G G     G  G   AGCCATTG A   A  GTTTTGCACTTTTC TTTTTCAATTG
     i                                                                         i

TTTTTATTGTTT GGTTGTTGTTTTATGTTATTTGATTTTTATTTGTGTTTTGTGTAG
 F  L  L  F   G  C  C  F  M  L  F  D  F  Y  L  C  F  V  *
uuuuuAuuGuuuGGuuGuuGuuuuAuGuuAuuuGAuuuuuAuuuGuGuuuuGuGuAG
uuuuuAuuGuuuGGuuGuuGuuuuAuGuuAuuuGAuuuuuAuuuGuGuuuuGuGuAG

     A  G   GG  G  G    A G  A   GA     A   G G    G G AG........AGAGAGGAATTTTGTTT 2133
     A  G   GG  G  G    A G  A   GA     A   G G    G G AG
     A  G   GG  G  G    A G  A   GA     A   G G    G G AG
```

green highlighting shows OLD prediction, for example:

```
AuuG   AuuuACAuu
AuuGUUUA   ACAuu
```

ND9

```
AuGuuuAuAUuuuuAuGuuuAUUuCGuuuAuGuuuuuGuuuAAUUUU  AuuuuAUU GuuuGuGuuGuAG Au        CL predicted Edited
                    s                     s                   s
A G   A AT    A G   ATT CG   A G     G   AATTTTTTA    ATTTG   G G  G AG A         Sylvio
A G   A AT    A G   ATT CG   A G     G   AATTTT  A    ATTTG   G G  G AG A         CL Brener
A G   A AT    A G   GTT CG   A G     G   GATTTTT A    ATTTG   G G  G AGTA         Esmeraldo
                                               ii                      i

GAU GUUUUGuuuGuuuuGuuGAuuGuAGUuuuuuAuuuuuuuuAuuGuuuuGuuAGuuuuuuuuuGuuuu
                                                                 s
GATTGTTT G   G    G  GA  G AGT     A       A  G    G  AG         A
GATTGTTTG    G    G  GA  G AGT     A       A  G    G  AG         G
GATTGTTT G   G    G  GA  G AGT     A       A  G    G  AG         A
        i

AUU GUAU  GuuuuuAUUUUuuA  AuuuGU  GGuuuuuAUUUUuGu AUU GUuGuGAUUU    GuuAuu
                                   s                                s  s
ATTTG ATTTG     ATTT   ATTA   GTTTGG     ATTTT G  ATTTGT G GATTTTTTTA  A
ATTTG ATTTG     ATTTT  ATTA   GTTTGG     ATTTT G  ATTTGT G GATTTTTTTG  A
ATTTG ATTTG     ATTT   ATTA   GTTTGA     ATTTTTGTTATTTGTTG GATTTTTTTG  G
                    i                         i ii      i

GGuuGAuuuuuGuG  GUUUuuGuuuuuGuC GuuuuAuGUUGuuGuAuAuuuuAuuuuGuuuAuuuuuGU    G
s
AG  GA     G GTTGTTTT G     G CTG    A GTTG  G A A    A    G   A     GTTTT G
GG  GA     G GT GTTT  G     G CTG    A GTTG  G A A    A    G   A     GTTTT G
GG  GA     G GTTGTTTT G     G CTG    A GTTG  G A A    A    G   A     GTTTTTG
               i    i                                                     i

uAuuCGuuuGuGuuuuGUUUuGuGUuGuuuGuuuAuuuuuuuuGGuuuGuGuuuuAGuuuuAGuuGuuuuuGuuA
         s           s        s
 A  CG   G G    GTTTTG GTTG   G   A        GG   G G    AG    AG   G     G  A
 A  CG   G G    GTTT G GT G   G   A        GG   G G    AG    AG   G     G  A
 A  CG   A G    GTTT A GT G   A   A        GG   G G    AG    AG   G     G  A
                    i    i

uACAUUUuuAUUAUU A GAA    C   GuGAAuGuuuuGAUUU GuuuGGUUUuuAuuuuGUU G  GuAAuG
                                              s    s
 ACATTT  ATTATTTATGAATT  CTTTG GAA G    GATTTTG   GGTTTT A    GTTTGTTG AA G
 ACATTT  ATTATTTATGAATTTTCTTTG GAA G    GATTTTG   GGTTT  A    GTTTGTTG AA G
 ACATTT  ATTATTTATGAATT  CTTTG GAA G    GATTT A   GATTT  A    GTTTGTTG AA G
                       ii                     i         i

AuGuuuACAU      C GuuuAuuuGuuG  AuuGAUuuuuuGuuGGuuuuuuuuuGuuGAAAuGuuAuC   C
       s
A G    ACATTT   CTG   A   G  GTTA  GAT     G  GG        G  GAAA G  A CTT C
A G    ACATT    CTG   A   G  GTTA  GAT     G  GG        G  GAAA G  A CTTTC
A G    GCATTTTTTCTG   A   G  GTTA  GAT     G  GG        G  GAAA G  A CTT C
            iiii                                                     i

     GUUAUUU   G GUUuAuuuGuGuuAuuAuuuuGuAuAuuAuuGGA    G  G    A  AAU  AAu
     s  s                                                              s
CTTT GTTGTTTTTTGTGTT A   G G  A  A    G A A  A  GGATTTTGTTGTTT ATTAATTTAA
     GTTATTTT  GTGTT A   G G  A  A    G A A  A  GGATTT GTTGTTT ATTAATTTAA
CTTTTATTATT    GTGTTTA   G G  A  A    G A A  A  GGATTTTGTTGTTTTATTAATTTGA
iiiii      iiii     i                                 i       i

AUGuACAuuuACAAuGuuGuuuuuGuuGuuGCAuACC AAuuuuuAuuuA   CAUuAuuuuAU  GUA A
                  s
ATG ACA   ACAA G  A     G  G  GCA ACCTAA    A   ATT CAT A    ATTTGTATA
ATG ACA   ACAA G  G     G  G  GCA ACCTAA    A   A   CAT A    ATTTGTATA
ATG ACA   ACAA G  G     G  G  GCA ACCTAA    A   ATTTCAT A    ATTTGTATA
                                                  iii
```

MURF5

```
s s    ss                   s       ss    ss   s       sss ss     s
ATATTGTTTTTGTTTTTATTTTATACTATTACAAATTTTTGGTTGTATAGG   TTTAAATTTAAATTTACATGT  Sylvio
 I  L  F  L  F  L  F  Y  T  I  T  N  F  W  L  Y  R     F  K  F  K  F  T  C
ATAATATATCTGTTCTTATTTTATACTATTACAAATTTTTGGTCGTATAAAAAACTTAAATTAAGATTTACATAT  CL Brener
 I  I  Y  L  F  L  F  Y  T  I  T  N  F  W  S  Y  K  K  L  K  L  R  F  T  Y
ATA   TTTTTGTTTCTATTTTATACTATTACAAACTTTTGGTCATATAAA   TTTAAATTACAACGTACATAT  Esmeraldo
 I     F  L  F  L  F  Y  T  I  T  N  F  W  S  Y  K     F  K  L  Q  R  T  Y
   iii                                             iii

      s      s  s       ss           s                  ss s        s  s
AATCATCTAAATAATTATATTGATGTATACAATATGACATTTTTTTGTTTAAATGGAGTGATGATTTGATTGAAT
 N  H  L  N  N  Y  I  D  V  Y  N  M  T  F  F  C  L  N  G  V  M  I  W  L  N
AGCCATTTAAATAATTATATTGATGCATACAATATGATATTTTTTTGTTTAAATGGGTTAATGATTTGGTTAAAT
 S  H  L  N  N  Y  I  D  A  Y  N  M  I  F  F  C  L  N  G  L  M  I  W  L  N
   CATTTAAATAGTTGTATTGATATATACAATATGATATTTTTTTGTTTAAATGGATTGATGATTTGGTTGAAT
    H  L  N  S  C  I  D  I  Y  N  M  I  F  F  C  L  N  G  L  M  I  W  L  N
iii

  s s   s      s s      s                      ss
ACTATACACATTAATATCATTATTATAAAACATTCATTTATAATATTAATAAATAATATTGAATATTTAATAATA
 T  I  H  I  N  I  I  I  I  K  H  S  F  I  I  L  I  N  N  I  E  Y  L  I  I
ACCATACACATTAATGTTATTATTGTAAAACATTCATTTATAATATTAGTAAATAATATTGAATATTTAATAATA
 T  I  H  I  N  V  I  I  V  K  H  S  F  I  I  L  V  N  N  I  E  Y  L  I  I
ACTACACATATTAATGTTATTATTATAAAACATTCATTTATAATATTGATAAATAATATTGAATATTTAATAATA
 T  T  H  I  N  V  I  I  I  K  H  S  F  I  I  L  I  N  N  I  E  Y  L  I  I

       ss     s      s s     s
TTATTTTATTT  ATTTAAAAAATTACTTTTAAAA GAATAG
 L  F  Y  L    F  K  K  L  L  L  K   E  *
TTATTTTATTTAAATT GAAAGAATACTTCTAAAA GAATAGA
 L  F  Y  L  N    W  K  N  T  S  K   R  I
TTATTTGC     ACT   AAAAATACTTTTAAAAAGAATAGGTTCGAGGGATTTTTA
 L  F  C      T     K  N  T  F  K  K  N  R  F  E  G  F  L
         iiii   iii                i
```

protein alignment:

. conserved in Tc; * conserved in Tc+Tb: < diff polarity Tc.

```
               .  ........... .   .   .  ...  .. ... ...... .....
Syl MURF5   1  ILFLFLFYTITNFWLYR-FKFKFTCNHLNNYIDVYNMTFFCLNGVMIWLN
CL MURF5    1  IIYLFLFYTITNFWSYKKLKLRFTYSHLNNYIDAYNMIFFCLNGLMIWLN
Esm MURF5   1  I FLFLFYTITNFWSYK-FKLQRTY-HLNSCIDIYNMIFFCLNGLMIWLN
Tb MURF5    1     MFLIHFVHYKTILQ--KYTFKFKHIFLSIDKYNSLFFNISGILIWLN
                   **             *      *    ** **  **   *  ****
                             <      <<              <


              . ... .. ....... ..........
Syl MURF5  75 TIHINIIIIKHSFIILINNIEYLIIILFYLFKKLLLKE*     87
CL MURF5   76 TIHINVIIVKHSFIILVNNIEYLIIILFYLNWKNTSKRI     88
Esm MURF5  73 TTHINVIIIKHSFIILINNIEYLIIILFCTKNTFKKNRFEGFL 89
Tb MURF5   74 IIHINIILIKYSFFILINNFEYLIIILIST              77
                ***  *  * ** ** ** ******
               <                          <<<<<<<<<
```

ND7, p.1

```
UAuCAuuuuAuGuuAuuuuuGGuAGuuuuuuuACAuuuGuAuCGuuuuACAuuuGG UC    CACAGC AuCCCGCAGCACAu     Tb edited
   H  F  M  L  F  L  V  V  F  L  H  L  Y  R  F  T  F  G         P  Q   H  P  A  A  H     Tb aa
                                                                 s         s
 A  A    A G  A     GG GG       ACA   A A CG TTTACA   GGTTC  TTTCACAGCTA CCTGCAGCCCAT     Sylvio DNA
 A  A    A G  A     GG GG       ACA   A A CG   TACA   GGTTC  TTTCGCAGCTA CCCGCAGCCCAT     CL Br. DNA
 A  A    A G  A     GG GG       ACA   A A CG TTTACA   GGTTCTTTTTCACAGCTA CCCGCAGCCCAT     Esmer. DNA
                                              ii           ii

   GGuGuuuuAuGuuGuuuAuuGuAuuuuuGu  GGuGA      AuuuAuuGuuuA U   AUUGA   uUGuAuuAuAGGuuAUUUGCAU
    G  V  L  C  C  L  L  Y  F  C    G  E        F  I  V  Y      I  D     C  I  I  G  Y  L  H

T  GG G    G G  G    A  A ATTTTTGTTTGG GA    TTA   A  G   ATC TTA TGATTTTTG A  A AGG  ATTTGCAT
TTTGG G    G G  G    A  A TTTTGTTTGG GA  TTTTA   A  G   ATC TTA TGATTTTTG A  A AGG  ATTTGCAT
T  GG G    G G  G    A  A ATTTTTGTTTGG GATTTTTTA   A  G   ATCTTTA TGA  TTTG A  A AGG  ATTTGCAT
 ii                       i             iiii                i        ii

CGUGGUACAGAAAAGUUAUGUGAAUAUAAAAGUGUAGAACAAUGUCUUCCGuAUUUCGACAGG UUAGAuuAuGuu
 R  G  T  E  K  L  C  E  Y  K  S  V  E  Q  C  L  P  Y  F  D  R  L  D  Y  V
                       s  s     s   s       s  s     s     s   s
CGTGGTACAGAAAAGTTATGTGAGTACAAAACAATAGAGCAGTGTTTACCA ATTTCGATAGGTTTAGA  ATG
CGTGGTACAGAAAAGTTATGTGAATATAAAACAATAGAACAGTGTTTGCCG ATTTTGATAGGTTTAGA  ATG
CGTGGTACAGAAAAGTTATGTGAGTATAAAACAGTAGAGCAGTGTTTACCA ATTTTGATAGATTTGGA  ATG


A   GuGuuuGuuGuAAuGAGCAuuuGuuGuCuuuAUGuuuuGAGuAuAuGuuGCGAuGuuGuuuGuCGuuAC GuuGu
    S  V  C  C  N  E  H  L  L  S  L  C  F  E  Y  M  L  R  C  C  L  S  L   R  C
                      s      s     s
ATTTG G   G  G AA GAACG   G  G C   G G    GAA A A G  GCGG G  A   G CA  AC G  G
ATTTG G   G  G AA GAACA   G  G C   A G    GAA A A G  GCGG G  A   G CA  AC G  G
ATTTG G   G  G AA GAACA   G  A C   A G    GAA A A G  GCGG G  A   G CA  ACTG  G
                                                                        i

GCAuuuAuGCGuuuAuuAAuuGuAGAAuuuACC    CGuAGuuuuAAuGGuuuGuuGuGuAuAuCAuG  uAuGGuuuuG
 A  F  M  R  L  L  I  V  E  F  T      R  S  F  N  G  L  L  C  I  S  C    M  V  L
               s
GCA   A GCG   G   AA  G AGAA   ACCTTTTCG GG    AA GG   A  A G A A CA GTTTA GG    G
GCA   A GCG   G   AA  G AGAA   ACCTTTTCG GG    AA GG   A  A G A A CA GTTTA GG    G
GCA   A GCG   A   AA  G AGAA   ACCTTTTCG GG    AA GG   A  A G A A CA GTTTA GG    G


GAuuuAGGuuGuuuGuCUCCGuuGUUAuGAuCAuuuGAG  GAA   CG  UGACAAAuuGAuGACAuuuuuuGAuuuA   uGU
 D  L  G  C  L  S  P  L  L  W  S  F  E    E     R    D  K  L  M  T  F  F  D  L     C
     s                                                    s
GA   AGG  G   A CTCCA  A  A GA CA   GAGTTGAATTTCGTTTGATAAG  AA GACA      GA   G TTTG
GA   GGG  G   A CTCCA  A  A GA CA   GAGTTGAA TTCGTTTGATAAG  GA GACA      GA   GTTTTG
GA   GGG  G   A CTCCA  A  A GA CA   GAGTTGAATTTCGTTTGATAAG  GA GACA      GA   G TTTG
                                                 i                            i

UGuGGuuGuCGuAuGCAuuuGGC UUUCAuGGuuuuAuuAGGuAUUCUUGAUGAuuuuGuuuuuGGuuuuGuuGAu
 C  G  C  R  M  H  L  A   F  M  V  L  L  G  I  L  D  D  F  V  F  G  F  V  D

 G GG  G CG A GCA   AGCTTTTCA GG    A  GGG A  CTCGA GA    G     GG    G  GA
 G GG  G CG A GCA   AGCTTTTCA GG    A  GGG A  CTCGA GA    G     GG    G  GA
 G GG  G CG A GCA   AGC TTTCA GG    A  GGG A  CTCGA GA    G     GG    G  GA
                       i

uuuuuGuuGuuGuuGAUAAuAuCAuGuuuGuuuGuuAuGGAuuGuuA        uGAuuuGuuAuuuGuG  GGuAA    UC   GuuuA
 F  L  L  L  L  I  I  S  C  L  F  V  M  D  C  Y          D  L  L  F  V    G  N         R  L
             s                                                                            s
     A  A  A YAA AA A CG G   G   G  A GGA  G  A    TTTTTTGA   G  G   G G  GG AATTTTTC   G   A
     A  A  A TGA AA A CG G   G   G  A GGA  G  A    TTTTTTGA   G  G   G G  GG AA  TTTC   G   G
     A  A  A TGA AA A CG G   G   G  A GGA  G  ATTTTTTTTTGA   G  G   G GTTGG AA     CTTTG   A
                                                 iii                    ii      iiiii iii

uuuUAuuuGCGuuuGCGuGGuuuGuCAuuuuuuGAuuuAuAuGAuuuA  GuuuuuA A   UAGuuuAAGuGGuGuuuuG
 F  Y  L  R  L  R  G  L  S  F  F  D  L  Y  D  L    V  F   N     S  L  S  G  V  L
                                      s                                         s
    A   GCG  ACG GG   A CG     GA TTA A GA TTGTTG    ATATTTTAG  AAG GG G    A
    A   GCG  ACG GG   A CG     GA TTG A GA TTGTTG    ATATTTTAG  AAG GG G    G
    A   GCG  ACG GG   A CG     GA TTA A GA TTGTTG    ATATTTTAG  AAG GG G    A
```

ND7 p.2

```
uCuCGuuCGuuAGGuAuGGuGuGAGAuuG  UCGuuuAuuuAGuuGuuA  UGA  GuUGuAuuuuAuGuuuuGuuAuGAu
 S  R  S  L  G  M  V  W  D  C    R  L  F  S  C  Y    E    L  Y  F  M  F  C  Y  D
                                                            s
 CCCG  CA  AGG A GG A GRGA TGTTTCG   A   AG  G  ATTTGA TA TATA  TTA G    G  A GA
 CCCG  CA  AGG A GG A GGGA TG TTCG   A   AG  G  ATTTGATTA TATATTTTA G    G  A GA
 CCCG  CA  AGG A GG A GGGA TGTTTCG   A   AG  G  ATTTGATTA TGTATTTTA G    G  A GA
                             i                         i       ii

uAuuGuuuuuGuuuuAuA GGuGAuGCAuuuGA   UCGuuuAuuuuACGuuuGuuuGAUA     uGCGuAuGAGuuuGuuG
 Y  C  F  C  F  I   G  D  A  F  D     R  L  F  L  R  L  F  D       M  R  M  S  L  L
                                           s                                    s
 A  G     G    A ATGG GA GCG   GATTTTCG    A     ACG   A   GA A  TTTTGCG A GAG   G  A
 A  G     G    A ATGG GA GCG   GATTTTCG    A     ACG   A   GA ATTTTTTGCG A GAG   G  A
 A  G     G    A ATGG GA GCG   GA TTTCG    G      CG   A   GA A TTTTTGCG A GAG   A  A
                                 i              i              ii

AuuuGuAAG   CAAuGuuuuuuuGuuGGuuuuuuuGuuuuuGGuuuuGuuuGuuuGuuuGAuuAuuuAuAuuGuGA     u
 I  C  K     Q  C  F  F  V  G  F  F  V  F  G  F  V  C  L  F  D  Y  L  Y  C  D
                        s
ATTTGTAAATTTCAA G       G  GG       GTTTTTGG    G   G   G   GA  A   G A  G GATTTT
ATTTGTAAATTTCAA G       G  GG       GTTTTTGG    G   G   G   GA  A   G A  G GATTTTT
ATTTGTAAATTTCAA G       A  GG       G    TGG    G   G   G   GA  A   G A  G GATTTTTT
                                     iiii                                     ii

AuuACCAuuG   AGACCAuuAuuAuGuuAuuuuAuAGuuuGuGGuGuuGuuGuuuGCCGGGuAuAUC AuuuGCUUG U
 I  T  I     E  T  I  I  M  L  F  Y  S  L  W  C  C  C  L  P  G  I  S   F  A  C
s             s                                         s
A AACCA  G TTAGACCA  A  A G  G    A AG   G GA G  G  G   GCCCGG A ATCTA   GCTTG T
A AACCA  GTTTAGACCA  A  A G  G    A AG   G GA G  G  G   ACCCGG A ATCTA   GCTTG T
G AACCA  G TTAAACCA  A  A G  G    A AG   G GA G  G  G   ACCCGG A A CTA   GCTTGTT
          i                                                       i           i

GuuGAACACCCC AAA       GGuGA    GuA    uuGuuuGuuAuuA UGuuuuuGuGuuGGuuuAuGuuCUCGuuuACGuuuGCGu
 V  E  H  P   K          G  E      Y      C  L  L  L   C  F  C  V  G  L  C  S  R  L  R  L  R
       s                                                s
G  GAACACCCCTAAG     TTGG GATTTTG A TTTTTG   G  A  GTTG     G G TGG   A GTTCCCG   ACG   ACG
G  GAACACCCC AAGTTTTTTTGG GATTTTG ATTTTTTG   G  A  GTTG     G G TGG   A GTTCCCG   ACG   ACG
G  GAACCCCCC AAG    TTTGG GATTTTG A TTTTTG   G  A  ATTG     G G TGG   A GTTCCCG   ACG   ACG
            i   iiiii              i

uGuGCGGAuuuuuuGCA    UA UUUGuuuAuuGGAuGuuuGuuuGCGuGGuuuuuuAuuGCAuGAuuuAGuuGC     CGuu
 C  A  D  F  L  H       I  C  L  L  D  V  C  L  R  G  F  L  L  H  D  L  V  A       V
                                                          s
 G GCGGA      GCA TTTTATTTTG   G  AGA G   G   ACG GG      A  GCA GA   AG  GC TTTTCG
 G GCGGA      GCATTTTTTA   TG  G  AGA G   G   ACG GG      A  GCA GA   AG  GC TTTTCG
 G GCGGA      GCA  TTTATTTTG   G  AGA G   G   ACG GG      G  GCA GA   AG  GCTTTTTCG
                 ii    iii                                                   i

uuAG GuAAuAuuGAuGuuGuuuuuGGAuCCGUAGAUC  GuuA  GuuuuAuAuGuG
 L   G  N  I  D  V  V  F  G  S  V  D    R  *
  s                                s
  AGTG AA A TGA G  G     GGA CCG AGACCTTG  A TG TTTA A G A
  GGTG AA A TGA G  G     GGA CCG AGATCTTG  A TG TTTA A G A
  AGTG AA A TGA G  G     GGA CCG AGACCTTG  ATTG TTTA A G A
                                            i
```

COIII p.1

```
AuGuuuuuGuuuC    GuuGuAuAuuuGuuGGuGuuA     GuGGuGuuuuuGuuuuuuuAuCuuuACCCGCCAuuGuuA    UU  CL Brener RNA
 M  F  L  F      R  C  I  F  V  G  V       S  G  V  F  V  F  L  S  L  P  A  I  V      I   CL Brener aa

A G     G   C TTTG  G A A   G  GG G  ATTTTTG GG G     G       A C   ACCCGCCA  G  A TTTTT  Sylvio DNA
A G     G   CTTTTG  G A A   G  GG G  ATTTTTG GG G     G       A C   ACCCGCCA  G  A TTTTT  CL Brener DNA
A G     G   CTTTTG  G A A   G  GG G  A  TTTG GG G     G       A C   ACCCGCCA  G  ATTTTTT  Esmeraldo DNA
             i                        ii                                          i

GuGuAuuGAuuGAuuuuGuuuAuuA       GGuuuuAuGuGuuuAUUAUUUG        GGuGuuuuGuuuuuGuuGAuGuAuGuuUU
 V  Y  W  L  F  C  L  L          G  F  M  C  L  L  F          G  C  F  V  F  V  D  V  C  F

G G A  GA  A    G   A  ATTTTTTTTGG    A G G   A  A   G   TTTTTGG G    G     G  GA G A G
G G A  GA  A    G   A  A   TTTTTGG    A G G   A  A   GTTTTTTTTGG G    G     G  GA G A G   TT
G G A  GA  A    G   A  A   TTTTTGG    A G G   A  A   G  TTTTTTGG G    G     G  GA G A G  TTT
                        iii                           iii                               iii

AuuuuuuuuuuuGuAuGuuuGuuAuuuuGuuuGuuAGuuuuAuuuGuGGAuuuAuuuuGuGAuuuuuuACGuGGu
 I  F  F  F  V  C  L  L  F  C  L  L  V  L  F  V  D  L  F  C  D  F  L  R  G
              s
A           G A G   G  A    G   G  AG    A   G GGA   A    G GA      ACG GG
A           G A G   G  A    G   G  AG    A   G GGA   A    G GA      ACG GG
A           G G G   G  A    G   G  AG    A   G GGA   A    G GA      ACG GG

AuuuuuGAuuuuuGuuGuuuAuuAC G   UGuuGUC  CA AuAuUGuuuuAuuuGAuuuGuuAuUA     GuGAAuuuAuuuuA
 I  F  D  F  C  C  L  L   R     V  V    Q   Y  C  F  I  W  F  V  I       S  E  F  I  L
      s                s
A     CA     G  G   A  ACTGTTTTG  GTC TCATA A TG    A   GA    G  A  A TTTTG GAA    A    A
A     GA     G  G   A  AC GTTTTG  GTCTTCATA A TG    A   GA    G  A  A TTTTG GAA    A    A
A     GA     G  G   A  GC GTTTTG  GTC TCATA A TG    A   GA    G  A  ATTTTTG GAA    A    A
                         i           i                              i

uuuAuGGCAuuuuuuACCGuAGuuuuuGGuuuAuGuuuAuuuuuAuGuuGuGA    GuuuGC   UUUUGuuuuuuGuuuA
 F  M  A  F  F  T  V  V  F  G  L  C  L  F  L  C  C  E      F  A     F  V  F  C  L

   A GGCA      ACCG AG     GG    A G    A     A G  G GA TTTG   GC TTTTTTG      G   A
   A GGCA      ACCG AG     GG    A G    A     A G  G GA TTTG   GCTTTTTTTG      G   A
   A GGCA      ACCG AG     GG    A G    A     A G  G GATTTTG   GC TTTTTTG      G   A
                                                     i         i

CCAuuAuuAuuuuGuuGuuuAuuAuGuGAuuAuGGuuuuGuuuuuuAuuGGuAuuuuuuAGAuuuGuuuAAuuuG
 P  L  L  F  C  C  L  L  C  D  Y  G  F  V  F  Y  W  Y  F  L  D  L  F  N  L
                                 s                               s
CCA  A  A    G  G   A  A G GA  A AG    G      A  GG A      AGA   A  TAA   G
CCA  A  A    G  G   A  A G GA  A GG    G      A  GG A      AGA   G  TAA   G
CCA  A  A    G  G   A  A G GA  A GG    G      A  GG A      AGA   A  TAA   G

uuGAuAAAuACAuuuuA UUUAuuuGuuA G CGGuuuAuuuGuuAAuuuuuGuuuGuuuuG  UUUUUGAuuuAGGuuu
 L  I  N  T  F  Y   L  F  V   S  G  L  F  V  N  F  C  L  F  C  F  W  F  R  F
  s                                   s
  AA AAA ACA    ATTTTA   G  ATGTCGG   G   G  AA     G   G    GTTTTTTTGA    AGG
  GA AAA ACA    ATTTTA   G  ATGTCGG   A   G  AA     G   G    GTTTTTTTGA    AGG
  GA AAA ACA    ATTTTA   G  ATGTCGG   G   G  AA     G   G    G TTTTTTGA    AGG
                                                              i

uuuuGuuG    UUGuuGuuuuGuAuuGuGAuuGAGuuuAuuAuuuGGuuuuuuAuuuuuGuGAAACC   AAuuGUGGG    AA
 F  C  C      C  C  F  V  L  W  L  S  L  L  F  G  F  L  F  L  W  N     Q  L  W      E
                                 s                                         s
    G  G  TTTTG  G    G A  G GA  AAG   A  A   GG      A     G GAAACC   AA  GTGGG TTTAA
    G  G  TTTTG  G    G A  G GA  GAG   A  A   GG      A     G GAAACC   AA  GTGGG TTTAA
    G  GTTTTTTG  G    G A  G GA  AAG   A  A   GG      A     G GAAACCTTTAA  ATGGGTTTTAA
        ii                                                          iii         i

uuuACAuuAuuAuuuGuuACA  UUAAGuuGuGGuGuuuuuGGuuCuAuuuuAuuuuuAA UUGAuuuA  UUACAuuuu
 F  T  L  L  F  V  T    L  S  C  G  V  F  G  S  I  L  F  L   I  D  L    L  H  F

   ACA  A  A   G  ACATTTTAAG  G GG G     GG   C A    A     AATTTGA   ATTTTACA
   ACA  A  A   G  ACA TTTAAG  G GG G     GG   C A    A     AATTTGA   ATTTTACA
   ACA  A  A   G  ACATTTTAAG  G GG G     GG   C A    A     AATTTGA   A TTTACA
                     i                                               i
```

COIII alignment, p.2

```
ACACAuGuuuuuuuAGGuGuAuuuuuGuuGuuuAuuuGUUUUGG UCGuuuAuuuAAuuuuuuGuGuAuGGAuACA
 T  H  V  F  L  G  V  F  L  L  F  I  C  F  G   R  L  F  N  F  L  C  M  D  T
                    s
ACACA G       AGG G A     G  G   A   GTTTTGGTTCG   A   AA      G G A GGA ACA
ACACA G       AGG G A     G  G   A   GTTTTGGTTCG   A   AA      G G A GGA ACA
ACACA G       AGG G G     G  G   A   G TTTGGTTCG   A   AA      G G A GGA ACA
                                      i

CGuuuuGuuuuuuuuGuAuGuuGuuuGuuuGuAuuGACAuuuuGuuGAUUGuAuuuGAuuuuuuuuuAuuGCGAuuu
 R  F  V  F  L  Y  V  V  C  L  Y  W  H  F  V  D  C  I  W  F  F  L  L  R  F
                             s s                                    s
CG    G       G A G  G   G   A G  GACA    G  GATTG A   GA        A  ACGA
CG    G       G A G  G   G   G A  GACA    G  GATTG A   GA        A  GCGA
CG    G       G A G  G   G   A A  GACA    G  GATTG A   GA        A  GCGA

GuuuAuuuuGAuGuuuuAuG   CGuuAuGuAuuuGuGuGuAuAAuuAuAuuuAUUUUUAUUUAUAUUUGUCUUUAAG
 V  Y  F  D  V  L  C     V  M  Y  L  C  V  *
            s
G   A    GA G    A GTTTCG  A G A   G G G A AA  A G   A     A
G   A    GA G    A GTTTCG  A G A   G G G A AA  A A   A     A
G   A    GA A    A GTTTCG  A G A   G G G A AA  G G   A
```

Cyb

```
AuGuuuCGuuGuAGAuuuuuAuuAuuuuuuuuuAuuAuuuAGAAAuuuGuGuuGUCUUUUAAUGUCAG              Tb edit
                                                                       s
A G   CG  G AGA     A  A        A  G   AGGAA   G G  GTCTTCTAATGTCAGGTTGTTTA       Sylvio
A G   CG  G AGA     A  A        A  G   AGGAA   G G  GTCTTCTAATGTCAGGTTGCTTA       CL Brener
A G   CG  G AGA     A  A        A  G   AGGAA   G G  GTCTTCTAATGTCAGGTTGTTTA       Esmeraldo

                    s  s     s   s s                             s  s     s
TATAGGATATATGGAGTGGGGTTCAGTTTAGGTCTATTTATTATGTTACAGATAATATGTGGTGTTTGCTTAGCATGAATG
TATAGAATATATGGAGTGGGGTTTAGTTTAGGTTTTTTTATTATGTTACAGATAATATGTGGTGTCTGTTTAGCGTGAATG
TATAGAATATATGGAGTGGGATTTAGTTTGGGTTTTTTTATTATGTTACAGATAATATGTGGTGTTTGTTTAGCATGAATG

     s     s     s           s        s        s     s           s  s  s     s
TTTTTTAGTTGTTTTATTTGTGCAAATTGATATTTTATTTTATTTTTGTGAGACTTTGATTTAGGCTTTGTGATTAGAAGT
TTTTTTAGTTGTTTTATTTGTGCAAATTGATATTTTATTTTATTTTTGTGAGATTTTGATTTAGGTTTTGTAATTAGAAGT
TTTTTCAGTTGCTTTATATGTGCAAATTGGTATTTTATCTTATTTTTATGAGATTTTGATTTAGGTTTCGTGATTAGGAGT

s s     s     s  s  s  ss        s                         s   s s  s           s
ATTCATATCTGTTTTACTTCTTTGCTATATTTTCTATTATATGTGCATATATTTAAATGTATAATATTGGTAATTTTATTT
ATTCATATTTGTTTCACTTCCTTATTATATATTTTTATTATATGTGCATATATTTAAATGTATAATATTGGTAATTTTATTC
GTACACATCTGTTTTACCTCATTATTATATTTTTTATTATATGTGCATATATTTAAATGCATAGTGTTAGTAATTTTATTT

  s  s     s     s      s        s     s    s  s     ss                s  s
GACACACATTTATTAGTATGATTTATAGGATTTATATTATTTTATGTTTATAATAATTATTGCATTTATTGGATACGTGTTA
GACACACATTTATTAGTGTGATTTGTAGGATTTGTATTATTTTATGTTCATAATAGTTATTGCATTTATTGGATATGTGTTA
GATACGCATTTGTTAGTGTGATTTGTAGGATTTATATTACTTATATTCATAATCATTATTGCATTTATTGGGTATGTGTTA

  s                  s    s        s     s           s     s  ss ss s  s        s
CCATGTACAATGATGTCTTATCGAGGATTAACAGTTTTTAGTAATATTTTAGCTACTGTTCCTGTATTCGGCCAGTGATTA
CCATGTACAATGATGTCTTATTGAGGATTAACAGTTTTTAGTAATATTTTAGCAACTGTACCTGTGATCGGTCAGTGATTG
CCGTGTACAATGATGTCTTATTGAGGGTTAACAGTCTTTAGCAATATTTTAGCAACTGTTCCCATATTTGGTCAGTGATTA

           s  s                 s        s              s     s  s  ss s  s
TGTTATTGAATATGGGGTAGTGAATTTATAAATGATTTTACGCTTTTAAAACTTCATGTATTACATGTCTTGCTCCCTTTT
TGTTATTGAATATGAGGTAGTGAATTTATAAATGATTTTACGCTTTTAAAACTTCACGTATTACACGTTCTACTTCCTTTT
TGTTATTGAATTTGAGGTAGTGAATTTATAAACGATTTTACTCTTTTAAAACTTCATGTATTGCATGTTCTGCTCCCTTTT

  s      s s           s              s                    s               s
GTTTTAATAATGGTATTATTTTTACATCTTTTTTGTTTACATTATTTTATGAGTTCAGATGCTTTTTGTGATAGGCTTGCT
GTATTAATAATGGTATTATTTTTGCATCTTTTTTGTTTACATTATTTTATGAGTTCAGATGCTTTTTGTGATAGGTTTGCT
GTTTTAATATTAGTATTATTTTTACATCTTTTTTGTTTGCATTATTTTATGAGTTCAGACGCTTTTTGTGATAGGTTTGCT

                    s                    s              s        s   s
TTTTATTGTGAGCGACTTTGCTTTTGTATGTGATTTTATTTAAGAGATATGTTTCTTGCATTTTTCATACTATTTTGTGTA
TTTTATTGTGAGCGACTTTGTTTTTGTATGTGATTTTATTTGAGAGATATGTTTCTCGCATTTTTCATATTATTTTGTGTA
TTTTATTGTGAGCGACTTTGTTTTTGTATGTGATTTTATTTGAGAGATATGTTTCTCGCATTTTTTATATTATTTTGTGTA

  s  ss                      s                    s              s           s
ATATATGTTATTTTTATAAATTGATATTTTGTTTTCCATGAAGAATCTTGAGTCATTGTTGATACATTAAAAACATCCGAT
ATGTACGTTATTTTTATAAATTGATATTTCGTTTTCCATGAAGAATCTTGGGTCATTGTTGATACATTAAAAACATCCGAT
ATGTATATTATTTTTATAAATTGATATTTTGTTTTCCATGAAGAATCTTGAGTCATTGTTGATACGTTAAAAACATCTGAT

        s  s           ss     s       s  s  s            s  s                  s
AAAATATTACCCGAATGATTTTTCCTATTTCTATTTGGTTTCCTTTAAAGCAGTGCCTGACAAATTTATGGGATTATTTCT
AAAATATTACCCGAATGATTTTTCCTATTTTTATTTGGCTTCCTT AAAGCAGTGCCTGACAAATTTATGGGATTATTTTT
AAAATATTGCCTGAATGATTTTTTTTATTTTTATTTGGTTTTCTC AAAGCAGTGCCCGATAAATTTATGGGATTATTTTT
                                             i

s   s    ss       s     s            s       s     s  s     s     s
AATGGTAGTTCTATTATTCGCATTATTTATGTTTATATTAAATTGCATATTATGGTTTGTATATTGCAGAAGTTCTTTATT
GATGGTAGTACTATTATTCGCATTATTTATGTTTATATTAAATTGCATATTGTGATTTGTTTATTGCAGAAGTTCTTTATT
GATGATAGTATTATTATTTGCATTGTTTATGTTTATACTAAATTGTATATTATGATTTGTTTATTGTAGAAGTTCTTTATT

    s    s     s  s        s                                         s  s
ATGATTAACATATTCGTTGATTCTATTTTATAGTATATGAATGAGTGGTTTTTTAGCTTTATATGTGGTGCTCGCTTATCC
ATGATTAACATATTCATTGATTCTATTCTATAGTATATGAATGAGTGGTTTTTTAGCTTTATATGTGGTACTTGCTTATCC
ATGACTAACGTATTCGTTAATTCTATTTTATAGTATATGAATGAGTGGTTTTTTAGCTTTATATGTGGTGCTTGCTTATCC

                  s        s        s     s                       s
AATATGAATGGAATTACAATATTGAGTGTTATTATTATTTTTGTTAGTTGTATGTAGATTAGATTAG
AATATGAATGGAATTACAGTATTGAGTATTATTATTATTTTTATTAGTTGTATGTAGATTAGATTAG
AATATGAATGGAATTACAATATTGAGTATTATTATTGTTTTTATTAGTTGTATGTAGATTAGATTAA
```

ATPase6

```
AuGuuuuuAuuuuuuuuuuuGuGAuuuAUUUuGGuuGCGuuuAuuA   UUAuGAuuAuAuuAuuGuGuGuGAuCCAGG    Sylvio edited
 M  F  L  F  F  F  C  D  L  F  W  L  R  L  L     L  W  L  Y  Y  C  V  W  S  R     Sylvio aa
        s                          s                 s
A G     A          G GA   A TTTGG  GCG   A  A  TTTA GA  A A  A  G G G GA CCAGG    Sylvio DNA
A G     G          G GA   ATTTTGG  GCG   A  A  TTTA GA  A A  A  G G G GA CCAGG    CL Brener DNA
A G     A          G GA   ATTTTGG  ACG   A  ATTTTTA GG  A A  A  G G G GA CCAGG    Esmeraldo
                           i                 ii

uuGuGuuuuGuuGuGuAuuuuAA UUGuuuAuuAuuGAuAuuuGAuuuuuuAuuAUUUuGuuuAuuuGAuuuAuA U
 L  C  F  V  V  Y  F  N    C  L  L  L  I  F  D  F  L  L  F  C  L  F  D  L  Y
  s
  G G    G  G G A    AATTTG   A  A  GA A   GA       A  A TTTG   A   GA   A ATT
  G G    G  G G A    AATTTG   A  A  GA A   GA       A  A TTTG   A   GA   A ATT
  A G    G  G G A    AATTTG   A  A  GA A   GA       A  ATTTTG   A   GA   A ATT
                                                        i

UCGuuuAuuGGuuuGuGuuuAuuuuuAuuGuuAuGGuuuAuGuuAUuuAA UUUGuAuAGuuuAAUUuuAuAUuAu
 S  F  I  G  L  C  L  F  L  L  L  W  F  M  L  F  N    L  Y  S  L  I  L  Y  Y
 s            s                 s                              s
TCG   A  GG   G G   A     A  G  A GG   A G  A  TAATTTTG A AG   AATT  A AT A
TTG   A  GG   A G   A     A  G  A GG   A G  A  TAATTTTG A AG   GATT  A AT A
TTG   A  GG   G G   A     A  G  G GG   A G  A  TAATTTTG A AG   GATT  A AT A

uGuAuuAC   UUAUuuG    AAuuuG UAuuuAuuAUUUuGuAuuGuuuuuuuGuAuuA    UAuGuCAuuuuuAuuuuuAuuu
 C  I  T     Y  L      N  L  Y  L  L  F  C  I  V  F  L  Y  Y      M  S  F  L  F  L  F
                                                                      s
 G A  ACTTTTTA  TG TTTAA   GTTA   A  A TTTG A  G       G A  ATTTTTA G CA     A     A
 G A  AC   TTA  TGTTTTAA   GTTA   A  A TTTG A  G       G A  ATTTTTA G CA     A     A
 G A  AC   TTA  TG  TTAA   GTTA   A  A TTTG A  G       G A  A TTTTA G CG     A     A
        iii       ii                                         i

uGuuuuuuGuuAGAuuuuuuuuuuAuuuAAuAAuuuA   UUGGuuGGuGAuA    AuuuuA    UGGAuGuuuuuuuuuAUCCGu
 C  F  L  L  D  F  F  L  F  N  N  L      L  V  G  D      N  F      M  D  V  F  F  I  R
           s                   s                         s
 G      G  AGA         A   AA AA   ATTTTTGG  GG GA ATTTTA   ATTTTTGGA G        ATCCG
 G      G  GGA         A   AA AA   ATTTTTGG  GG GA ATTTTA   ATTTTTGGA G        ATCCG
 G      G  GGA         A   AA AG   ATTTTTGG  GG GA ATTTTG   ATTTTTGGA G        ATCCG

uuuuuAuuAuGuuuuuuGGAAuGuuuuuCuuuAuuGuGuCGuuGuuuGuCAACAuuuuuACGuuuGUUUUGuAAu
 F  L  L  C  F  L  E  C  F  S  L  L  C  R  C  L  S  T  F  L  R  L  F  C  N

     A  A G      GGAA G     C   A  G G CG   G   G CAACA     ACG   GTTTTG AA
     G  A G      GGAA G     C   A  G G CG   G   G CAACA     ACG   GTTTTG AA
     G  A G      GGAA G     C   A  G G CG   G   G CAACA     ACG   GTTTTG AA

uuAuuGuCA    UCCCAUUUuuuAuuGuuG  AuGuuuuuuGAUUUUuuAuAuuuuuuuuuuGuuuuuuuuAuuuuuuuu
 L  L  S      S  H  F  L  L  L    M  F  F  D  F  L  Y  F  F  F  V  F  F  I  F  F

  A  G CA TTTTCCCA   TTTA  G  GTTA G     GA  TTTTA A          G        A
  A  G CA TTTTCCCA  TTTTA  G  GTTA G     GA  TTTTA A          G        A
  A  G CATTTTTCCCA   TTTA  G  GTTA G     GA  TTTTA A          G        A
         i          i

uGuuuuuuAGAuuuuuuA      GuGuuuAuuuuAuuuAuuuuuGuGuuuuGuuuuuGuuuAuuGuuuuA     CGGuuuuuuG
 C  F  L  D  F  L        V  F  I  L  F  I  F  V  F  C  F  C  L  L  F  Y       G  F  L
                                                              s  s         s         s
 G      AGA      ATTTTTTG G   A    A   A     G G    G     G   A  G    A    TCGG      G
 G      AGA      ATTTTTTG G   A    A   A     G G    G     G   G  G    A TTTTTGG      A
 G      AGA      A TTTTTG G   A    A   A     G G    G     G   G  A    ATTTTTTGG      A
                  i                                                    iiii

uAuuuAuuGGAuuuGuuuGCA     GCCAuAuuGC  AGuuAuuuAuuuuuuuGuAAuAuGAuuuuACAAuuGAuAAuGGAu
 Y  L  L  D  L  F  A       A  I  L    Q  L  F  I  F  C  N  M  I  L  Q  L  I  M  D
                    s
 A   A  GGA   G   GCA TTTTGCCA A  GCTTAG  A   A      G AA A GA    ACAA  GA AA GGA
 A   A  GGA   G   GCGTTTTTGCCA A  GCTTAG  A   A      G AA A GA    ACAA  GA AA GGA
 A   A  GGA   G   GCA TTTTGCCA A  GCTTAG  A   A      G AA A GA    ACAA  GA AA GGA
                     i

uuuuuGuuAuuuuuGuuAuuuuGuuAGuuuuGCGAuuuuAuuuuuuAuAGuuuGuuGuAuGuuuAuuuuuGuA
 F  L  L  F  L  L  F  C  *

     G  A     G  A    G  AG
     G  A     G  A    G  AG
     G  A     G  A    G  AG
```

MURF1

```
AAATCCTTGTTTTTATATATCATACACATATTTATATTTCTTATTATCTACAGCTTTATTATCTTATGTGATTAT          T.b. DNA
 K  S  L  F  L  Y  I  I  H  I  F  I  F  L  I  I  Y  S  F  I  I  L  C  D  Y           T.b. pep
       s      s s           s  s           s  s
AAATCCTTTTGTTATTATATATTTTATACATATCTTTATTTTTTTGATACTTTATAGTTTATTATCTTATGTGATT          Sylvio DNA
AAATCCTATTGTTACTATATTTTATACATATCTTTATTTTTTTAATACTTTATAGTTTATTATCTTATGTGATT            CL DNA
AAATCCTATTGTTATTGTATTTTATACACATTTTTATTTTTTTAATCCTTTATAGTTTATTATCTTATGTGATT            Esmo DNA

ACTACACTAACATTACTAAGCTTTGATTTACTCTGATTATTAATTATTAACCTATTTTGAATAACATTATTAGAT
 T  T  L  T  L  L  S  F  D  L  L  W  L  L  I  I  N  L  F  W  I  T  L  L  D
 s  s   s ss                 s             ss       s    s s
ACATCAATATAACATTAATTACCTTTGATCTATTGTGATTAACGGTTATTAATGGATTTATTGTATTATTTTTAGAT
ATATTAATGTGGCATTAATTACCTTTGATCTATTGTGATTAACGGTTATTAACGGATTTATTGTATTATTTTTAGAT
ATATTAATATATCATTAATTACCTTTGATTTATTGTGATTAACATTTATTAATGGATGTGTTGTATTATTTTTAGAT

TCTTATATTTGTTTCATCTTTATACTCTTATTCTTATTTTGCTTTACATTATTTTTTTGCTTTCTATCATTCGAT
 S  Y  I  C  F  I  F  I  L  L  F  L  F  C  F  T  L  F  F  C  F  L  S  F  D
              s  s  s                                         s           s
TCATATATATGCTTTATATTTTTAATTTTATTCCTTTTTTGTTTCTTTTTTATGTTTTGTTTTCTTACATTTGAC
TCATATATATGCTTCATCTTCTTAATTTTATTCCTTTTTTGTTTCTTTTTTATGTTTTGTTTCCTTACATTTGAT
TCATATATTTGCTTCATCTTTTTAATTTTATTCCTTTTTTGTTTCTTTTTTATGTTTTGTTTTCTTACATTTGAT

ACCAGATTTTTATTCATTATTATTATTATTCAATACATTATTATATTCTTATTTATATTTATTAATCATATTATT
 T  R  F  L  F  I  I  I  I  I  Q  Y  I  I  I  F  L  F  I  F  I  N  H  I  I
        s                  s       s         s        s       s     s     s
ACAAGATTTTTATTTATGATAATTATTATTCAATACATTATTATTATATTATTTTTATTTGTAACACATATAATTTTT
ACAAGATTTTTATTTATGATAATTATTCTTCAATACATTATTATTATATTATTTTTATTTGTTACACACATAATTTTT
ACAAGATTCTTATTTATGATAATTATTATTCAATATATTATTATTTTATTATTTCTATTTGTTACACATATAATCTTT

ATTATATCAATCCTCTTTGAAATATTTAGCTTATTGCTCTTTTTATTATTAATGTCCAGTAGATTTGGATATAAA
 I  I  S  I  L  F  E  I  F  S  L  L  L  F  L  L  L  M  S  S  R  F  G  Y  K
                  s        s ss    s   s                   s
ATATCAATTTTATTTGAACTTTTTAGTTTACTACTTTTCTTACTTTTATTATCAAGTCGCTTTGGATATAAAATA
ATATCAATTTTATTTGAACTTTTTAGTCTTCTACTCTTCCTACTTTTATTATCAAGTCGTTTTGGATATAAAATA
ATATCAATTTTATTTGAAATTTTTAGTCTTTTACTTTTCTTACTTTTATTATCAAGTCGTTTTGGATATAAAATA

ATTTTAGTTCTATGATATTACTACATGTTAAACTTAATAAATTTTATATTATTATTTTATATTATTATATTTTATG
 I  L  V  L  W  Y  Y  Y  M  L  N  L  I  N  F  I  L  L  F  I  L  L  Y  F  M
        s  s        s        s                        s  s s
TTAATTTTATGATATTATTATATGATTAACTTAATTAATTTTATTCTATTATTTTTATTGCTTTACTTCTTACTC
TTAATTTTATGATATTATTATATGATTAACTTAATTAATTTTATTCTATTATTTCTACTGCTTTACTTCTTACTC
TTAATTTTGTGGTATTATTACATGATTAATTTAATTAATTTTATTCTATTATTTTTACTACTTTACTTCTTACTC

ATATTAAACTACTGTTTTTTCCTATGTGATTTTTGTTTTTTAGTTTTTGATGAAGAATGACTTGGAATTTTATGC
 I  L  N  Y  C  F  F  L  C  D  F  C  F  L  V  F  D  E  E  W  L  G  I  L  C
  s     s     s           s        s           s                 ss     s
CTGCACTTTTGCTTCTTCCTATGTGATTTTTGTTTTTTAATTTTTGATGAAGAATGGATGGGTGTAATTTGCTTA
CTTCACTTTTGCTTCTTCCTATGTGACTTTTGTTTCTTAATTTTTGACGAAGAATGGATGGGGGTGATTTGCTTA
CTCCACTTCTGCTTTTTCCTATGTGATTTTTGTTTTTTAATTTTTGATGAAGAATGGATGGGTGTGCTTTGCCTA

CTATTTTACACATTATTAATTTTATTCAAATTATACATCGCCTTTTTAATTTTGTTTATGGAGCAACTTTATATT
 L  F  Y  T  L  L  I  L  F  K  L  Y  I  A  F  L  I  L  F  M  E  Q  L  Y  I
        s         s          s        s     ss s                 s
TTTTTTATCATATTAATTTTATTTAAATTATATATAGCGTTTTTAATTTTATTTATGGAACAATTATATATCAGA
TTTTTTATAATATTAATTTTATTTAAATTGTATATAGCATTTTTAATCTTATTTATGGAACAATTGTATATCAGA
TTTTTTATTATATTAATTCTATTTAAATTATATATAGCATTTTTGGTGTTATTTATGGAACAATTATATATCAGA

AGACTTGGTGTCTTTATTTTTATTTATATGTTAACTTTTTATATTTTGTTTTGTTTTATATTAATAATTCTACTA
 R  L  G  V  F  I  F  I  Y  M  L  T  F  Y  I  L  F  C  F  I  L  I  I  L  L
  s   s s   s    s                 s   s    s     s           s
TTGGGAGTCTTTGTTTTCATTTATATGTTAACTTTCTATATTTTGTTTTGTTTTATTTTAATTATTTTACTAATA
TTAGGAGTCTTTGTTTTCATTTATATGTTAACTTTTTATGTTTTATTTTGCTTTATTTTAATCATTTTACTAATA
TTAGGAATTTTTATTTTTATTTATATGTTAACTTTCTATATTTTATTTTGCTTTATTTTAATTATTTTACTAATA

ATTAGCTTCATTTATTTTTATATTTTATTCATCAAACTTCTATTATTTCAATCTTGTACATGCGTTTTAATTGGA
 I  S  F  I  Y  F  Y  I  L  F  I  K  L  L  L  F  Q  S  C  T  C  V  L  I  G
  ss ss    s  s         s           s             s        s  s        ss
TGCTTAGTCTATTTCTATATTATATTTATAAAACTTGTTTTATTACAATCCTGTACTTGCGTTATAATTGGAATA
TGCTTAGTCTACTTCTATATTATATTTATAAAACTTATTTTATTACAATCATGTACTTGTGTCATAATAGGATTA
TGTCTGATCTATTTTTATATTATACTTATAAAACTTGTTTTATTACAATCCTGTACTTGCGTTATAATAGGGTTA

TTAAACAGCTTTGCTATCGTTAGTTTACTATTTGTGTTAAGTGTGAATAATTTTTGCTTTTTATTCCTAATATTT
 L  N  S  F  A  I  V  S  L  L  F  V  L  S  V  N  N  F  C  F  L  F  L  I  F
  s  s           s  s                                s   s     s       s ss
AATAGCTTTGCAATTATAAGTTTACTCTTTGTACTTAGTGTAAATAATTTTTGTTTTTTATTTCTAATTTTCATC
AATAGTTTTGCAATTATAAGTTTACTCTTTGTACTTAGTGTAAATAATTTTTGCTTTCTATTTTTAATTTTTATC
AACAGTTTTGCAATTATGAGCTTACTCTTTGTACTTAGTGTAAATAATTTTTGCTTTCTATTTTTAATTTTTATT
```

MURF1 p.2

```
ATTTCAACAAAAAATTATATCTTTTATTTATATTTAAATTTTCACTTAATTTATAGTATAAGTTTAGTATTACTT
 I  S  T  K  N  Y  I  F  Y  L  Y  L  N  F  H  L  I  Y  S  I  S  L  V  L  L
  s       s   s  s     s        s              s     s      s s  ss  s
TCCACAAAAAATTACATCTTTTATTTATACCTCAATTTTCATTTAATTTATAGCATAAGTCTATGCATTTTATTA
TCCACAAAAAATTACATCTTTTACTTATACCTCAATTTTCATTTAATCTATAGTATAAGTCTGTGTATTCTATTA
TCAACAAAAACTTATATTTTTTACTTATACCTTAATTTTCATTTAATTTATAGCATAAGTTTATGTGTTTTATTA

ATTATTATATACTATTTTTTTATTATATATAATATTTTTGACTTTAAATACAATGAAAACTATTTTTTAATCAAC
 I  I  I  Y  Y  F  F  I  I  Y  N  I  F  D  F  K  Y  N  E  N  Y  F  L  I  N
           s        s                       s     s        s     s        s
ATTTTATATTACTTTTTTTTGATTTACAATATTTTTGATATTAAATATAACGAAAATTACTTTCTTATAAATTTC
ATTTTATATTATTTTTTTTTAATTTACAATATTTTTGATATTAAGTATAACGAAAATTATTTTCTCATAAATTTT
ATTTTATATTATTTTTTTTATAATTTACAATATTTTTGATATTAAATATAATGAAAATTACTTTCTCATAAATTTT

TTCATATTTTTTTCTTTTTTTAATAACTTTTTAATAAGTTTACTACTTGCATGTCTCTTTCTTTGCATTGGAGCT
 F  I  F  F  S  F  F  N  N  F  L  I  S  L  L  L  A  C  L  F  L  C  I  G  A
  s                    s  ss s        s     s  s  s        s        ss s  s
ATCTTTTTTTCATTTTTTAATAATTTTATACTTAGTATTATGTTGTCTTGCTTATTTTTATGTATAGGTACAATT
ATCTTTTTTTCATTTTTTAATAATTTTTTGCTTAGTATCATGTTATCTTGCTTATTTTTATGTATAGGTACTATC
ATTTTTTTTTCATTTTTTAATAACTTCTTACTTAGTATTATGTTATCCTGTTTATTTTTGTGTATAGGCTCTATT

ATTCCAATTGTATTTGGATTTTTTATCAAAGTTTTCTGTCTATTATTACAACTAAGTTACCTTTGTATTTGTATT
 I  P  I  V  F  G  F  F  I  K  V  F  C  L  L  L  Q  L  S  Y  L  C  I  C  I
     s           s                    ss             s  s  s   s    s  s
CCCATTGCCTTTGGTTTTTTTATAAAAGTTTTTTGCTTGTTAACACAACTTAGCTATATTGGTATTTGCATTATT
CCCATTGCCTTTGGTTTCTTTATAAAAGTTTTTTGCTTACTAACACAACTTAGCTACATTGGTGTTTGCATCATT
CCCATCGCCTTTGGTTTCTTTATAAAAGTTTTTTGCTTACTAACACAACTTAGTTACATCGGTATTTGTATTATT

GGCTTTTTCTTTATAATATGACTTATTATCATTTATATTTTTTATTTTAGATTAATTGTTAATATATTTATTTTT
 G  F  F  F  I  I  W  L  I  I  I  Y  I  F  Y  F  R  L  I  V  N  I  F  I  F
           s  s      s  s s        s                       s     s
TTATTTTTAATAATTTGACTTGTTATAATTTATATCTTTTATTTTAGACTTATTATTAATATTTTCATTTTTTCA
TTATTTTTAATCATTTGACTTATTATAATTTATATTTTTTATTTTAGACTTATTATTAACATTTTTATTTTTTCA
TTATTTTTAATTATCTGACTTGTTGTTATTTATATTTTTTATTTTAGACTTATTATTAACATTTTTATTTTTTCA

TCTTATCAATTTCTTGGTTTTTGAGTTGTAAAATTATCTTTTATCAATATTAAAAATTTATTATTCTTCATTTGT
 S  Y  Q  F  L  G  F  W  V  V  K  L  S  F  I  N  I  K  N  L  L  F  F  I  C
       s         s    s s           s ss s        s     s       s         s
TATCAATTTATTGGATTCTGAGGAATTAGACTTCATATGGTTTGCTTTAATAATTTGATTTTTTGTTGTTGTTGT
TATCAATCTATTGGATTTTGAGTAGTTAGACTTCATTTAGTTTGCTTTAATAATTTAATTTTTTTTTGTAGTTGT
TATCAATTTATTGG-TTTTGGGTAATTAGACTTCATTTAATCTGCTTTAACAATTTAATTTTTTTTTGTAGTTGC
              i
TCTAGCGTATATATACTTTTCTTTGATATTATTAACTTATTTGATCTTATTCTATAACT
 S  S  V  Y  I  L  F  F  D  I  I  N  L  F  D  L  I  L
         s  s s                                      s     s
TCTTTTTATGCATTATTTTTTGATATTATT         AATTTATTTGATCTCATTTTATAA
TCTTTTTATACATTATTTTTTGATATTATT         AATTTATTTGATCTCATTTTATAA
TCTTTTTATGCACTGTTTTTTGATATTATTTACAAAATTAATTTATTTGATCTTATTTTGTAA
                              iiiiiiiii
```

CR3

```
ATGTTTGA   TTGTTTGGTTTTG TTGTTTTTTTATTGTTTGTTTGTACATTTTTTTTGTTTTTTATTTGTTTGTG     AT   Tb edit
                       s           s                                  s
A G   GATTTTTG   AA    GTTTA TTTTTTG  A   G   G ACA        G   TTTA   G   G G TTTTA    Sylvio
A G   GATTTTTG   AA    GTTTA  TTTTTG  A   G   G ACA        G   TTTA   G   G G TTTTA    CL Brener
A G   GATTTTTG   AA    ATTTA  TTTTTA  A   G   G ACA        G  TTTTA   A   G GTTTTTA    Esmeraldo
                             i                                 i              i

TTGTTTTTATGTTTGTTATTTAGTTTTTGTTTTTTATTGGATTTTTGTTTTTTATTTAATA TGGGTTTA TTGTTG
                            s
  G     A G   A  A   GG     A      G  GGA     G      G   AA ATTGGG   ATTTG  G
  G     A G   A  A   GG     A      G  GGA     G      G   AA ATTGGG   ATTTG  G
  G     A G   A  A   GG     G      G  GGA     G      G   AA A TGGG   ATTTG  G
                                                             i

TGTTTATTTTTTTTTTTTATTTTATCATTTGA    TATGTGTATCA   AATTGTTATTTATTATTTAG

 G   A            A    A CA   GATTTTTA G A A CGTTTAA  G  A   A  A   AG
 G   A            A    A CA   GATTTTTA G A A CGTTTAA  G  A   A  A   AG
 G   A            A    A CA   GATTTTTA G A A CGTTTAA  G  A   A  A   AG
```

ND1

```
                     s     s     s s  s            s
Syl.dna      1 TTGTTAGTACATCTAGATATAATTATTGTTTTTGTTGTTTTTATTTTAGTTTTATTAATCTTATCAGGATTTGTT  75
CL.dna       1 TTGTTAATACATTTAGATGTTATTATTGTTTTTGTTATTTTTTATTTTAGTTTTATTAATCTTATCAGGATTTGTT  75
Esmo.dna     1 TTGTTAATACATTTAGATATTATCATTGTTTTTGTTATTTTTTATTTTAGTTTTATTAATCTTATCAGGATTTGTT  75

                    s        s   ss   s      s           s s  s  s     s           s     s
Syl.dna     76 AGCCTTTGTGAAAGGAAAGCTTTGGCTATTGTACAACTTAGAGTCGGGCCAGCTCTTTTTTTATTTGGGATATTG 150
CL.dna      76 AGCCTTTGTGAAAGAAAAGTTTTAGCTATTGTACAACTTAGAGTTGGGCCGGCTCTTTTTTTATTTGGAATATTA 150
Esmo.dna    76 AGCCTCTGTGAAAGAAAAATTTTAGCTATTATACAACTTAGAATCGGACCAGCTCTCTTTTTATTTGGAATATTG 150

                       s     s  s                     s    s   s       s
Syl.dna    151 ACACCTATTACAGACGGCATTAAACTTTTTGTTAAATTTACTTTATTTGTAATTGGTTTTGATGGTATTTACTTT 225
CL.dna     151 ACACCTATCACAGATGGTATTAAACTTTTTGTTAAATTTACTTTGTTTATAATTGGCTTTGATGGTATTTACTTT 225
Esmo.dna   151 ACACCTATTACAGATGGTATTAAACTTTTTGTTAAATTTGCTTTATTTGTAATTGGATTTGATGGTATTTACTTT 225

                    s  s  s ss                 s                  s s  s     s
Syl.dna    226 CTTTTCGGATTATTAATAACATTATTCTGCATATTTTTTCCATGATTCTTCTTACCTTTAGGGTTTATTATTCTT 300
CL.dna     226 CTTTTTGGGTTATCAATAACATTATTCTGCATTTTTTTTCCATGATTCTTCTTACCTTTAGGATTTATTATTCTT 300
Esmo.dna   226 CTTTTTGGATTGTTGATAACATTATTCTGCATTTTTTTTCCATGATTCTTCATTCCCTTAGGATTTATTATTCTT 300

                    s   s s     s  s  s  ss  s     s                   s        s       ss
Syl.dna    301 TTTGATAAAAGCTTTTCCATCTTGTTTTTATTAAGTATACACTTAGTTTGTAATGTGTTTAGTGTTTTTTTAGAT 375
CL.dna     301 TTTGACAAAGGATTTTCCATTTTGTTTCTACTAAGTATACACTTAGTTTGTAATGTATTTAGTGTGTTTTTAGTT 375
Esmo.dna   301 TTTGATAAAGGATTTTCTATTTTATTCTTATTAAGTGTACACTTAGTTTGTAATGTATTTAGTGTTTTTTTAGTC 375

                             s        s               s s     s                    s
Syl.dna    376 GGATGCTTTTTATTTTCTAGTTGTTTTGTTTATTTAGCAACTATGCGTACATTATTTTTTTCGATTATCTCAGAA 450
CL.dna     376 GGATGCTTTTTATTCTCTAGTTGTTTTGTTTATTTAGCAACCATGCGTACATTATTTTTTTCGATTATTTCAGAA 450
Esmo.dna   376 GGATGCTTTTTATTTTCTAGTTGCTTTGTTTATTTAGCAGCCATGCGCACATTATTTTTTTCGATTATTTCAGAA 450

                    s                       s   s s          s   s        s     s  s  s  s
Syl.dna    451 AGTGCCTTACTTATTTTACTTTATTGCTTATATCTGCTAGATTTTTATAGTTTTTTTGGTATTAAGGATTTGTGT 525
CL.dna     451 AGTGCTTTACTTATTTTACTTTATTGCTTTTATTTACTAGATTTTTATAGTTTTTTTGGTATTAAAGACTTGTGT 525
Esmo.dna   451 AGTGCTTTACTTATTTTACTTTATTGCTTTTATTTACTAGATTTTTTTAGCTTTTTTGGAATTAAAGATTTATGC 525

               s    s           s                 s  sss s        s    s  s  s   s s
Syl.dna    526 GTTAACCAACTATCATTATTTAATTGTTTTTTATTAGGAACTTTATTTATCGTAACTTTTTGAATTACATTACTC 600
CL.dna     526 GTTAACCAACTATCATTGTTTAATTGTTTTTTATTGGGGATTCTATTTATCGTAACTTTTTGGATTGCATTACTC 600
Esmo.dna   526 ATTAATCAACTATCATTGTTTAATTGTTTTTTATTAGGACTTTTATTTATCATAACATTCTGAATTACGTTACTC 600

                 s     s      s s  s              s           s  s     s  s   s    s
Syl.dna    601 TGCGATGGGTTGAGACTACCATTTGATTATATGGAATGTGAAAGTGAATTAGTTGCAGGACTTATAACAGAATTA 675
CL.dna     601 TGCGATGGCTTGAGATTGCCATTTGATTATATGGAGTGTGAAAGTGAGTTAGTTGCAGGGCTTGTAACGGAATTA 675
Esmo.dna   601 TGTGATGGCTTGAGATTACCGTTTGATTATATGGAATGTGAAAGTGAATTGGTTGCTGGACTTATAACAGAATTA 675

                 s  s  s                       s        s         s       s   s    s
Syl.dna    676 TCGGGTGTTTTCTTTGTTATTTACTCAGTTTTAGAAATAACGCATTTACTCTTATGTACAATTTTGCTTGCTGGG 750
CL.dna     676 TCTGGCGTATTCTTTGTTATTTACTCAGTTTTGGAAATAACGCATTTACTCGTATGTACGATTTTACTTGCTGGA 750
Esmo.dna   676 TCTGGTGTATTCTTTGTTATTTACTCAGTTTTAGAAATAACACATTTACTCTTATGTACAATCTTATTTACTGGG 750

               s          s  ss s   s          s                       s  ss          s
Syl.dna    751 CTATGTATTGGTGGCTTGTTTGTTTGCTTTAAGTCTATATTAATACTCATAATTTGCTTTCTAATACCAAGAGTA 825
CL.dna     751 TTATGTATTGGTGGCTTATTTATTTGCTTTAAATCTATATTAATACTCATAATTTGCTTTCTAATACCAAGGGTA 825
Esmo.dna   751 TTATGTATTGGGGGGCTATTTATTTGCTTTAAATCTATATTAATACTCATAATTTGTTTCTTAATACCAAGAGTA 825

                           s                  s      ss                 s ss           s s
Syl.dna    826 ATATGTTTTAGGTTAAAAATTACTACTGCACGAACATTTGTACTACTTTTTCTTTTTATCATAGGATTTTTATTA 900
CL.dna     826 ATATGTTTTAGGCTAAAAATTACTACTGCACAAACATTTGTACTACTTTTTCTTTTTGTTATAGGATTTTTACTA 900
Esmo.dna   826 ATATGTTTTAGGTTAAAAATTACTACTGCACAAACATTCATACTACTTTTTCTTTTTGTTGTAGGATTTTTACTC 900

                     s s               s           s    s
Syl.dna    901 TTTTTACTAATAGCTGTTACAAAATTATTATGCTTACTTTTATAA 945
CL.dna     901 TTTTTATTAATAGCTGTTACAAAATTATTATGCTTATTTTTTTAA 945
Esmo.dna   901 TTTTTATTGATAGCTGTTACAAAACTATTATGCTTATTTTTTTAA 945
```

COII

```
                                         s   s s
mCOII Syl.dna      1 AAGAUUUAAAUUAUGAGUUUCAUUCUAUCAUUUUGAAUGAUAUUUUUAAUAGAUUCUGUUAUAGUGUUAUUAUCU  75
mCOII CL.dna       1 AAGAUUUAAAUUAUGAGUUUUAUUUUGUCAUUUUGAAUGAUAUUUUUAAUAGAUUCUGUUAUAGUGUUAUUAUCU  75
mCOII Esmo.dna     1 AAAAUUUAAAUUAUGAGUUUUAUUUUAUCAUUUUGAAUGAUAUUUUUAAUAGAUUCUGUUAUAGUGUUAUUAUCU  75
COII Tula.dna      1 AAGAUUUAAAUUAUGAGUUUUAUUUUGUCAUUUUGAAUGAUAUUUUUAAUAGAUUCUGUUAUAGUGUUAUUAUCU  75
                                 ^^^

                                      s     s       sss s  s            s    s        s
mCOII Syl.dna     76 UUUGUUUGUUUUGUGUGUGUGUGAAUAUGUGCACUACUUCUUUCUACAGUUUUAUUUGUUACAAAGAUAAAUAAU 150
mCOII CL.dna      76 UUUGUUUGUUUUGUGUGUGUGUGAAUAUGUGUGUUGCUCCUUUCUACAGUUUUAUUUGUUACAAAGAUAAAUAAU 150
mCOII Esmo.dna    76 UUUGUUUGUUUUGUGUGCGUGUGGAUAUGUGCAUUGCUUCUUUCUACAGUUCUAUUAGUUACAAAAAUAAAUAAU 150
COII Tula.dna     76 UUUGUUUGUUUUGUGUGUGUGUGAAUAUGUGUGUUGCUCCUUUCUACAGUUUUAUUUGUUACAAAGAUAAAUAAU 150

                                      s   s       s  s                 s                       s
mCOII Syl.dna    151 AUAUAUUGUACAUGAGAUUUUAUAUCAUCAAAAUUUGUGGAUACUUAUUGGUUUGUAAUAGGAGUCAUGUUUAUU 225
mCOII CL.dna     151 AUAUAUUGUACAUGAGACUUUGUAUCAUCAAAGUUUGUGGAUACUUAUUGGUUUGUAAUAGGAGUCAUGUUUAUU 225
mCOII Esmo.dna   151 AUAUAUUGUACAUGAGAUUUUAUAUCAUCGAAGUUUGUGGAUACUUAUUGAUUUGUAAUAGGAGUCAUGUUUAUC 225
COII Tula.dna    151 AUAUAUUGUACAUGAGACUUUGUAUCAUCAAAGUUUGUGGAUACUUAUUGGUUUGUAAUAGGAGUCAUGUUUAUU 225

                              s        s s   s    ss       s     s  s      s    s              s
mCOII Syl.dna    226 AUGUGUUUAUUAUUAAGACUAUGUUUAUUGCUAUAUUUCGGUUGCUUAAAUUUUGUUAGUUUUGAUUUAUGUAAG 300
mCOII CL.dna     226 AUGUGUUUACUAUUAAGAUUGUGUCUAUUGUUAUAUUUUGGUUGUUUGAAUUUUGUUAGUUUUGAUUUAUGUAAG 300
mCOII Esmo.dna   226 AUGUGUUUAUUAUUAAGACUUUGUUUAUUAUUAUAUUUUGGUUGUUUAAAUUUUAUUAGCUUUGAUUUAUGUAAA 300
COII Tula.dna    226 AUGUGUUUACUAUUAAGAUUGUGUCUAUUGUUAUAUUUUGGUUGUUUGAAUUUUGUUAGUUUUGAUUUAUGUAAG 300

                       s     s  s     s           s     s        s           s  s  s        s  s
mCOII Syl.dna    301 GUUGUAGGAUUUCAAUGAUAUUGAGUAUACUUUUUAUUUGGAGAAACUACAAUAUUCAGCAAUUUAAUACUUGAG 375
mCOII CL.dna     301 GUAGUAGGUUUCCAAUGAUAUUGAGUAUAUUUUUUAUUUGGAGAGACUACAAUAUUUAGCAACUUAAUACUUGAA 375
mCOII Esmo.dna   301 GUAGUAGGUUUUCAAUGGUAUUGAGUAUACUUUUUGUUUGGAGAAACUACAAUAUUUAGUAAUUUAAUACUCGAG 375
COII Tula.dna    301 GUAGUAGGUUUCCAAUGAUAUUGAGUAUAUUUUUUAUUUGGAGAGACUACAAUAUUUAGCAACUUAAUACUUGAA 375

                             s     s  s        ss    s  s     s              ss                s
mCOII Syl.dna    376 AGUGAUUAUUUGGUAGGUGAUAUGCGUCUAUUACAGUGUAACCAUGUACUCACAUUAUUAAGUUUAGUAAUUUAU 450
mCOII CL.dna     376 AGUGAUUACUUGGUGGGUGAUAUGCGUCUAUUGCAGUGUAAUCAUGUACUCACAUUAUUAAGUUUAGUAAUUUAU 450
mCOII Esmo.dna   376 AGUGAUUAUUUGGUGGGAGAUAUGCGCUUAUUACAAUGUAACCAUGUACUCACAUUGCUAAGUUUAGUAAUUUAC 450
COII Tula.dna    376 AGUGAUUACUUGGUGGGUGAUAUGCGUCUAUUGCAGUGUAAUCAUGUACUCACAUUAUUAAGUUUAGUAAUUUAU 450

                             s  s  s  s        s              s                 s
mCOII Syl.dna    451 AAAUUGUGAGUAUCAGCUGUAGAUGUUAUCCAUUCAUUUACAUUAGCUAGUUUAGGUAUUAAGGUAGA--G-A-A 521
mCOII CL.dna     451 AAAUUGUGAGUAUCUGCUGUAGAUGUUAUCCAUUCAUUUACGUUAGCUAGUUUAGGUAUAAAGGUAGA--G-A-A 521
mCOII Esmo.dna   451 AAAUUGUGGGUGUCAGCCGUAGAUGUAAUCCAUUCAUUUACGUUAGCUAGUUUAGGUAUAAAGGUAGA--G-A-A 521
COII Tula.dna    451 AAAUUGUGAGUAUCUGCUGUAGAUGUUAUCCAUUCAUUUACGUUAGCUAGUUUAGGUAUAAAGGUAGAuuGuAuA 525

                       s  s            s s        s     s  s                 s     s  s  s
mCOII Syl.dna    522 CCGGGGAGGUGUAAUGAAGUUAUUUUAUUCGCAUCUAACAAUGCAACAAUAUAUGGGCAAUGUAGCGAGUUAUGU 596
mCOII CL.dna     522 CCGGGUAGGUGUAAUGAAAUCAUUUUAUUUGCAUCUAAUAAUGCAACAAUAUAUGGGCAAUGCAGCGAGUUAUGU 596
mCOII Esmo.dna   522 CCAGGUAGGUGUAAUGAAAUUAUUUUAUUUGCAUCCAAUAAUGCAACAAUAUAUGGACAAUGUAGUGAAUUAUGU 596
COII Tula.dna    526 CCGGGUAGGUGUAAUGAAAUCAUUUUAUUUGCAUCUAAUAAUGCAACAAUAUAUGGGCAAUGCAGCGAGUUAUGU 600

                       s  s           s                                 s s       s
mCOII Syl.dna    597 GGGGUAUUACAUGGAUUCAUGCCAAUUGUUAUAUGUUUUAUAUAAAA-GGUGUAUAAUCUACAGAUAUAAAAAGUG 671
mCOII CL.dna     597 GGGGUCUUACAUGGAUUUAUGCCAAUUGUUAUAUGUUUUAUAUAAAA-GGUAUAUAAUCUACAGAUAUAAAAGGGG 671
mCOII Esmo.dna   597 GGUGUAUUACAUGGAUUUAUGCCAAUUGUUAUAUGUUUUAUAUAAAAAGGUAUGUAAUCUAUAUAUAUAAA-UGUA 671
COII Tula.dna    601 GGGGUCUUACAUGGAUUUAUGCCAAUUGUUAUAUGUUUUAUAUAAAA-GGUAUAUAAUCUACAGAUAUAAUUAAAA 675
                                                               ^^^   ^^^^^^^^^^^^^^
                                                               TER     COII gRNA
```

Fold-back base-pairing:

```
                          TER
          5'-GTTTAGGTATAAAGGTAGAuuGuAuACCAGGTAGGTGTAATG
                    ||||   |||||||.||.|||   || .|.
    AGAATTAGGGTGAAAAATATAGACATCTAATATGTGGAAAATATATTTTG-5' Sylvio
                 TTAATATAGACATCTAATATATGGAAAATATATTTTG-5' Tulahuen
                           ^^^^^^^^^^^^^^
                              COII gRNA
```

Tulahuen sequence Genbank# L22643

MURF2

```
AuGuuuGGuuGuuuuA----AuuuAGuuuuAuuuuuAuGUUUUGAUUGuGUCGUGUUUUUGAUUUAUUAUGUGUU       SW predicted editing
                                       s                         s       v
A GT  GG  G    ATT  A   AG    A     A GTTTTGATTGAGTCGTGTTTTTGATTTATTATGTGAT       Sylvio DNA
A G   GG  G    ATT  A   AG    A     A GTTTTGATTGAGTCGTGTTTTTGATTTATTATGTGTT       CL Brener DNA
A G   GG  G    ATTTTA   AG    A     A GCTTTGATTGAGTCGTGTTTTTGATTTGTTATGTGTT       Esmeraldo DNA
                  ii
   s v  s           s                          s           s           s  s
AGAACCTACGATTTTATTTTATGATGATTTGATTTAGATTATATATTATATGATTTTGTATTTGATTTTGTGGTG
AGAGCATACGATTTTATTTTGTGATGATTTGATTTAGATTATATATTGTATGATTTTGTATTTGATTTTGTGGTA
AGAGCATATGATTTTATTTTGTGATGATTTGATTTAGATTATATATTATATGATTTTGTGTTTGATTTTGTAGTA

  s     s  s     s     s        v     ss       v  s                     s
TGTATAACATTCATATTCATATTCATTTTAGGTTTTTTTGTTAGAATATTTTTTAGTTTTGTATTTGTATTATTA
TGCATAACATTTATATTTATATTTATTTTAGGTTTTTTCGTTAGAATCTTCTTTAGTTTTGTATTTGTATTATTA
TGTATAACGTTCATATTTATATTTATTTTAGGGTTTTTTATTAGAATTTTTTTTAGTTTTGTATTTGTATTACTA

              s   sss  ss    s                  s  s  s        s  s
TTTATAACATTTTTTGGTACATATACATTGTCTATGTTATATA    CAGGTTATTATATATTCTATATATATAT
TTTATAACATTTTTCGGTACATACACATTGTCTATGTTATATATATACAGGTTACTATATATTTTACATATATAT
TTTATAACATTTTTTGGTGTGTATGCATTATCTATGTTATATA    CGGGCTATTATATATTTTATATATATAT
                                           iiii
      s     s        s     sv s    s     s      s  s          s          s
ATTATACAATTTTATATGTTTCTTTTTCACA  TTCGGTATCAATTTTTTGATATATTATTTAGAATTTTTTACG
ATTATATAATTTCATATGTTTCTTTTTTACG  TTCGGTATTAATTTTCTGATATATTATTTGGAATTTTTTACG
ATTATACAATTTTATATGTTTTTTTTTTTCGCATTTGGTATCAATTTTTTGGTATATTATTTGGAATTTTTTATG
                               ii
           v   s s        s   v                   s               s s  v  s
TTTATATTATTATATTTATTTTTTGATTTTATAAGTTTTTCATGTTTTATTTATAATTTTTTTGGTCTATTTTTT
TTTATATTATTTTATCTGTTTTTTGATTTTATAAGTTTTTCATGTTTTATTTATAATTTTTTTGGTCTATTCTTC
TTTATATTATTTTATTTATTTTTTGACTTTTTAAGTTTTTCATGTTTTATCTATAATTTTTTTGGTTTGTTATTT

        s  s                       s                 s      s    s
ACATTTAACATCATATTTTGTACGTATTTGTTTTGTTTATTTTATTTTATAATCATTTTTCTATTTTGTTTTATA
ACATTTAATATCATATTTTGTACGTATTTGTTTTGCTTATTTTATTTTATAATTTTTTTTTTATTCTGTTTTATA
ACATTTAATATTATATTTTGTACGTATTTGTTTTGTTTATTTTATTTTAT         TTTATTTTGTTTTATA
                                                  iiiiiiiii
           v            s          s  s        s  s     s     s  s        s
TTTTTTGTAATACGTTGTTTATTTATAATTGTATTTGATTTTTTATTCTTCAATTTTGATATCTTCATATCATTA
TTTTTTGTAATACGTTGTTTATTTGTAATTGTATTCGACTTTTTATTTTTTAATTTCGATATTTTTATATCATTA
TTTTTTGTAATTCGTTGTTTATTTGTAATTGTATTTGATTTTTTATTTTTTAATTTTGATATTTTCATATCATTG

        s  ss        s       s  v  s  s        s     s        v
ACGTTATGTGATATACAGTATGTAGATTTCGTATGCTTCTTAATATTATATTTTAATTATATTACAAATTTCATA
ACGTTATGTGATGTACAGTATATAGATTTCGTGTGTTTCTTAATATTGTATTTTAATTATATAACAAATTTCATA
ACGTTATGCGACATACAGTATGTAGATTTTGTTTGTTTTTTAATATTATATTTCAATTATATCACAAATTTCATA

     v           s              ss          s        s   s    s        s
TATGGTTTTTATTGTTTTATAATTATATTAGGATTAATTTTTTTATTATTATATATGGTACTTAATTTATACTTT
TATGGATTTTATTGTTTCATAATTATATTAGGGTTAATTTTTTTATTATTATACATGGTACTTAATTTATATTTT
TATGGCTTTTATTGTTTTATAATTATATTAGGACTAATTTTTTTGTTATTATATATGATACTCAATTTATATTTT

  v         v    v  s   s  v     vs s s    s      s  s  s
GGATTTATTTTTTTAATCTACGGACTAATATCTTTTTTAATAAATTATATATTTTGAATATATATAATATATAGT
GGTTTTTTTTTTATAATTTATGGATTAATATCTTTTCTGATAAATTATATATTCTGAATATATATAATATATAGT
GGATTT TTTTTATAATATATGGATTACTATC ACTTTAATAAGTTATATGTTCTGGATATATATAATATATAGT
      i                         i
        s  ss  s s     s  v        v        s                 v  s        s
AGAAGTTGTTATATATTAATGCCATCTATTTTAATATTTTTTAAGTTTATTTATTTTGATGTATTTTTAATATTT
AGAAGTTGTTATATATTAATGCCGTCTATTTTAATATTTTTTAAGTTTATTTATTTTGATGTTTTTTTAATATTT
AGAAGTTGCTACGTACTGATGCCATCAATTTTAATTTTTTTTAAATTTATTTATTTTGATGTATTCTTAATATTC

     s        s  s  s                        s           s    s  s
ATATTTATATTAATCTTATTTATAATATGTTTTTTTAGTTTTTTTTTAAAAGATTTTTTATTCTTGTCATTGTTT
ATATTTATATTAATTTTGTTCATAATATGTTTTTTTAGTTTTTTTTTAAAAGATTTTTTATTTTTGTCATTGTTT
ATATTCATATTAATTTTATTTATAATATGTTTTTTTAGTTTTTTTCTAAAAGATTTTCTATTTTTATCATTGTTT

              s   s s        s  s   sss     s ss  s       v v
TTTGATATATTTGGAGCATTATTTAATTATGACATATGTACATATTTTGTGTTTTATTTCAATTTACACCAATTT
TTTGATATATTTGGAGCACTGTTTAATTACGATATATACACATACTCTGTATTTTATTACCATTTACACCAATTT
TTTGATATATTTGGGGCATTGTTTAATTACGATATACACACATATTCCGTATTTTATTACCATTTACACCAATTT

  ss      s                   s  s
TGTATAACGCAATTATTATTTCTTTATACATAG
TGTGTAACGCAATTATTATTTCTTTATACGTAG
TGCATAACGTAATTATTATTTCTTTATACATAA
```

COI

```
                                              s    s                                   s
COI Syl.dna         1  AAAATATGTTTTTTTATTTGCCTTGTTTGCTTAAGTGTTTCCCACAAAATGATTGGGATTTGTTACCTTTTAGTT    74
COI CL.dna          1   GAATATGTTTTTTTATTTGCCTTGTTTGCTTAAGTGTTTCCCACAAAATGATTGGGATTTGTTATCTTTTAGTT    73
COI Esmo.dna        1 TTAAATATGTTTTTTTATTTGCCTTATTTGTTTAAGTGTTTCCCACAAAATGATTGGGATTTGTTACCTTTTAGTT    75
                            ^^^
                           s                 s  s           s           s  s     s  s     s     s
COI Syl.dna        75 GCTATCTTATGTGGATTTGTAGGTTATGTTTATTCACTCTTTATTAGATTAGAACTTTCATTGGTAGGTTGTGGA   149
COI CL.dna         74 GCTATATTATGTGGATTTGTAGGTTATGTTTATTCACTTTTTATTAGATTAGAACTTTCATTGGTAGGCTGTGGG   148
COI Esmo.dna       76 GCTATCTTATGTGGATTTGTAGGATACGTTTATTCACTCTTTATTAGATTGGAGCTTTCGTTAGTAGGCTGTGGG   150

                                 s        s        s   s s  s           ss             s
COI Syl.dna       150 GTTCTTTTTGGGGATTATCAGTTTTATAATGTTTTGATTACAGCACATGGGTTAATAATGGTTTTTGCATTTATT   224
COI CL.dna        149 GTTCTTTTTGGAGATTATCAATTTTATAACGTTTTGATTACAGCACATGGACTAATAATGGTTTTTGCATTTATT   223
COI Esmo.dna      151 GTTCTTTTTGGAGATTATCAATTTTATAATGTTCTAATCACAGCACATGGATTAATAATGGTTTTCGCATTTATT   225

                                    s  s  s           s                 s                    s
COI Syl.dna       225 ATGCCTGTTATAGTTGGAGGATTTGTTAATTATTTTGCACCTGTTATGGTTGGATTTCCTGATATGGTTTTCCCT   299
COI CL.dna        224 ATGCCTGTTATAGTGGGGGGATTTGTTAATTATTTTGCACCTGTTATGGTTGGATTTCCTGATATGGTTTTTCCT   298
COI Esmo.dna      226 ATGCCTGTTATAGTTGGAGGGTTTGTTAATTACTTTGCACCTGTTATGGTAGGATTTCCTGATATGGTTTTTCCT   300

                                                         s  s        s     s  s  s  s  s        s
COI Syl.dna       300 CGATTAAATAATATGAGTTTTTGGATGTTTATTGGGGGATTTGGTTGTCTTGTAAGTGGGTTTTTGACTGAAGAA   374
COI CL.dna        299 CGATTAAATAATATGAGTTTTTGGATGTTTATTGGTGGATTTGGTTGCCTTGTGAGCGGATTCTTAACTGAAGAG   373
COI Esmo.dna      301 CGATTAAATAATATGAGTTTTTGGATGTTTATTGGCGGGTTTGGTTGTCTTGTAAGTGGTTTTTTGACTGAAGAA   375

                        s              s   s    s                 s     s        ss             s
COI Syl.dna       375 GGCATGGGGGTTGGTTGAACACTATATCCTACTTTAATTTGTGTAGATTTTCATTCAAGCCTAGCTTGTGACTTT   449
COI CL.dna        374 GGTATGGGGGTTGGTTGGACATTATACCCTACTTTAATTTGTGTGGATTTCCATTCAAGTTTAGCTTGTGACTTT   448
COI Esmo.dna      376 GGTATGGGGGTTGGTTGAACATTATATCCTACTTTAATTTGTGTAGATTTTCATTCAAGTTTAGCTTGTGACTTC   450

                              s  s  s        s     s  s  s     s                        s
COI Syl.dna       450 GTTATTTTTTCGGTACACTTTTTAGGTATATCGAGCATTATAAATTCTATTAATATAATTGGAACTATTTTTTGC   524
COI CL.dna        449 GTTATTTTCTCAGTTCACTTTTTAGGTATATCAAGTATTATTAATTCTATTAATATAATTGGAACTATTTTTTGC   523
COI Esmo.dna      451 GTTATTTTTTCAGTTCACTTTTTGGGTATTTCTAGCATTATAAATTCTATTAATATAATTGGAACTGTTTTTTGC   525

                              s     s     s  s        s  ss s     s         s    s  s  s  s     s
COI Syl.dna       525 TGTAGAAGGAAATATTTTAGCTTCCTTATTTGGACGTTATTTATTTGAGGAAGCCTAGTCACGTCTGCGCTTTTG   599
COI CL.dna        524 TGTAGAAGAAAATACTTTAGCTTTCTTATTTGGACATTATTTATCTGAGGAAGCCTAGTTACGTCTGCACTTTTA   598
COI Esmo.dna      526 TGTAGAAGAAAATACTTTAGTTTTCTTATTTGAACACTGTTTATTTGAGGAAGCTTAGTTACATCAGCACTTTTA   600

                              ss s  s  ss s     s  s              s  s        s
COI Syl.dna       600 ATACTTACTTTACCAGTTCTAGCTGGGGGTGTTACATTACTTTTGTGTGATAGAAACTTTAATACATCATTTTAT   674
COI CL.dna        599 ATACTTACATTACCAGTCTTGGCTGGTGGTGTTACATTACTTTTGTGCGATAGAAACTTTAATACATCATTTTAT   673
COI Esmo.dna      601 ATACTTACACTGCCGGTCCTAGCTGGTGGCGTTACATTACTTTTATGTGATAGAAATTTTAATACATCATTTTAT   675

                              s  s  s  s  s     s  s     s     s                 s     s  s  s
COI Syl.dna       675 GATGTTGTGGGAGGTGGGGACTTAGTATTGTTTCAACATTTGTTTTGATTTTTTGGTCATCCTGAAGTTTATATT   749
COI CL.dna        674 GATGTTGTTGGGGGTGGAGACTTAGTGTTGTTTCAGCATTTATTTTGATTTTTTGGTCACCCTGAAGTCTATATT   748
COI Esmo.dna      676 GATGTTGTTGGAGGGGGTGATTTAGTATTATTTCAACATTTATTTTGATTTTTTGGTCATCCTGAGGTGTACATT   750

                            s             s        s    s      s        s        s           s
COI Syl.dna       750 ATTATATTACCTATTTTTGGATTAATTTCAACTACTCTTGAAGTTATTGGATTTCGATGCATTTTTAGCACAGTT   824
COI CL.dna        749 ATTATACTACCTATTTTTGGGTTAATTTCTACTATTCTTGAGGTTATTGGGTTTCGATGTATTTTTAGCACGGTT   823
COI Esmo.dna      751 ATTATATTACCTATTTTTGGATTAATTTCTACTATTCTTGAAGTTATTGGATTTCGATGCATTTTTAGCACGGTT   825

                        s                 ss             s  s  s        s  s
COI Syl.dna       825 GCAATGATATATTCTATGCTTCTAATTGCTATTTTGGGATTTTTTGTTTGAGCACATCATATGTTTGTTGTAGGT   899
COI CL.dna        824 GCAATGATATATTCTATGCTTTTAATTGCTATTTTAGGTTTTTTTGTTTGGGCACATCATATGTTTGTTGTAGGT   898
COI Esmo.dna      826 GCGATGATATATTCTATGCTGTTAATTGCTATTTTAGGTTTCTTTGTTTGAGCTCATCATATGTTTGTTGTAGGT   900

                                       s           s  s        s              s     s  s     s
COI Syl.dna       900 ATGGATGTTGATTCTAGAACTTACTTTGGGACTGTTACTGTTTTGATTGGATTACCTACATGTATTAAATTATTT   974
COI CL.dna        899 ATGGATGTTGATTCTAGGACTTACTTTGGAACCGTTACTGTTTTGATTGGATTACCCACATGCATTAAATTGTTT   973
COI Esmo.dna      901 ATGGATGTTGATTCTAGGACTTACTTTGGAACTGTTACTGTCTTGATTGGATTACCTACATGTATCAAATTGTTT   975

                           s        s   s                s     s        s        s              s
COI Syl.dna       975 AATTGAATGTATAGCTTTCTATACACAGATTTAATCATAACATTTGAAATCTATTTTGTGTATATGTTTGTTTTA  1049
COI CL.dna        974 AATTGGATGTATAGCTTTTTATACACAGATTTAATTATAACCTTTGAAATCTATTTTGTATATATGTTTGTTTTG  1048
COI Esmo.dna      976 AATTGGATGTATAGTTTTTTATACACAGATTTAATTATAACGTTTGAAATATATTTTGTGTATATGTTTGTTTTA  1050
```

```
                                  s  s     s  s  s                 s              s  s
COI Syl.dna    1050 ATGTTTCTTTTTGGTGGTGTTACAGGGTTGTTTTTATCAAATGTTGGGCTTGATATAATGCTGCATGATACTTAT 1124
COI CL.dna     1049 ATGTTTCTTTTTGGTGGGGTTACGGGGTTGTTTTTATCAAATGTTGGGCTTGATATAATGCTACACGATACTTAT 1123
COI Esmo.dna   1051 ATGTTTCTTTTTGGAGGAGTTACGGGTTTATTTTTATCAAATGTTGGCCTTGATATAATGCTACACGATACTTAT 1125

                            s  s  s        s   s s                    s  s     s     s  s
COI Syl.dna    1125 TTTGTAGTGGCACATTTTCATTATGTCCTATCTATTGGAGCTGTTGTTGGTTTTTTTGGTGGTTTTTTTCACTTT 1199
COI CL.dna     1124 TTTGTAGTAGCTCATTTTCATTATGTCTTGTCTATTGGAGCTGTTGTTGGGTTCTTTGGCGGTTTCTTTCACTTT 1198
COI Esmo.dna   1126 TTTGTAGTAGCACACTTTCATTACGTCTTATCTATTGGAGCTGTTGTTGGTTTTTTTGGAGGTTTCTTCCACTTT 1200

                      s        ss s   s    s     ss       s     s              s     ss    s
COI Syl.dna    1200 TTAATGAAATGACTTCCTATTGAACTTTACCTATTTTGAGAATTCTTTTTTATATGCTGTTTATGGGTTGGTGTA 1274
COI CL.dna     1199 TTGATGAAATGGTTGCCTATTGAACTTTATTTATTTTGAGAATTCTTTTTTATATGCTGCTTATGAGTTGGAGTA 1273
COI Esmo.dna   1201 TTGATGAAATGACTGCCTGTTGAGCTTTATTTATTTTGGGAATTTTTTTTTATATGCTGCTTATGGATTGGAGTA 1275

                        s   s           s  s  s  s                          s                 s
COI Syl.dna    1275 AATACGCTCTTTTTTCCTTTACACAGTTTGGGTATGTTTGCTTTTCCTAGAAGAATCTCTGATTATCCTATTAGT 1349
COI CL.dna     1274 AATATGCTTTTTTTTCCTTTACATAGTTTAGGTATGTTTGCTTTTCCTAGAAGAATTTCTGATTATCCTATTAGC 1348
COI Esmo.dna   1276 AATATGCTTTTTTTTCCTTTGCATAGCTTAGGTATGTTTGCTTTTCCTAGAAGAATTTCTGATTATCCTATTAGC 1350

                               s  s     s  s     s  s           s  s  s                    s  s
COI Syl.dna    1350 TTTTTATATTGAAGCTCCTTTAGTTTATATGGCATGCTGCTTATTACATCTCTAGTCTTATTTTGCTGTTGCTTA 1424
COI CL.dna     1349 TTTTTATATTGGAGCTCCTTCAGTTTATATGGTATGCTGCTTATCACATCCCTAGTCTTATTTTGCTGTTGTTTA 1423
COI Esmo.dna   1351 TTTTTATATTGAAGTTCCTTTAGCTTATACGGTATGCTGCTTATTACGTCTCTAGTCTTATTTTGCTGTTGCTTG 1425

                               s           s     s  s            s                      s
COI Syl.dna    1425 TTTAGTATTTTGTTCTTTTGAGATTATTGTTTGTTCTTTGTAAATCTATATGTATATAGTCTTTTTTGCTTTTTT 1499
COI CL.dna     1424 TTTAGTATTTTATTCTTTTGAGATTATTGCTTGTTCTTTGTAAATTTATATGTATATAGTCTTTTTTGTTTTTTT 1498
COI Esmo.dna   1426 TTTAGTATTTTGTTCTTTTGAGACTATTGCTTATTCTTTGTAAATTTATATGTATATAGTCTTTTTTGCTTTTTT 1500

                      s            s    s  s        s     s        s         s           s s
COI Syl.dna    1500 TACTTTAATTCTTGATTACCTTGTACTATGGGTTTATACTTACTTGTAGTAGATTTTACACATATTGTACTAGAT 1574
COI CL.dna     1499 TACTTTAATTCTTGATTACCTTGCACTATGGGATTATACTTACTTGTCGTAGATTTTACACATATTGTATTAGAT 1573
COI Esmo.dna   1501 TATTTTAATTCTTGACTACCCTGCACTATGGGATTATATTTACTTGTTGTAGATTTTGCACATATTGTATTGGAT 1575

                      ss     s s                               s         s  s  s        s  s  s
COI Syl.dna    1575 TATTTACTTCTAATTTATGTTTTTGCTTTGTTTTTTTTTATTTATTTTTGACACTCATTATTTTTATTCTTCTAT 1649
COI CL.dna     1574 TATTTACTTTTAATTTATGTTTTTGCTTTGTTTTTTTTTATTTATTTTTGACACTCTTTGTTTTTATTCTTCTAC 1648
COI Esmo.dna   1576 TACCTACTTTTGATTTATGTTTTTGCTTTGTTTTTTTTTATTTTTTTTTGACATTCTTTATTTTTATTTTTTTTAT 1650


COI Syl.dna    1650 GTATAAAGAAAT  1661
COI CL.dna     1649 GTATAAAGAAATA 1661
COI Esmo.dna   1651 GTATAAAGAA    1660
                       ^^^
```

CR4

```
                     s           s   s
AATTTGTGAAGGAGAAGAAGGGGGAGGTTTGGAAAGTAGAGAAG AGGGTTT CGAAAGATAGA  Sylvio DNA
AATTTGTGAAGGAGAAGAAGGAGGAGGTTTGGAGAGTAGAGAAG AGGGTTTTCGAAAGATAGA  CL Brener DNA
AATTTGTGAAGGAGAAGAAGGGGGAGGTT GGAAAGTGGAGAAGTAGGGTTTTCGAAAGATAGA  Esmeraldo DNA
                             i              i       i

s s                   s   s    s
GTTTTTTTTAAGTCTGTTTTGGGGG AAA  GGGGGAGAGG GGTTTGTTGTTTTTTTGTTTG
GTTTTT   AAGTTTGTTTTGGGGGTAAATTGGAGGAAAGG GGT  GTTGTTTTTTTG   G
GTTTT    AAATTTGTTT GGGGG AAATTGGGGGAAAGGTAGT  G  GTTT    GTTTG
     iiii          i     i   ii          i   ii ii    iiii iii

    ss        s s s                                    s   s
GGGTAAATTTGGAGAAGAGTTTGGGAGAATTTTGG  AATTGT  GTTTAAAGCGAAAAGA
GGGTAGATT GGAGGAAAGTTTGGGAGAATTTTGGT AATTGTT GTTTAAAGCGAAAAGA
GGGTGAATT GGAGGAGAATTTGGGAGAATTTTGGTTAATTGTTTGTTTAAAGCGGAAAAA
         i                         ii      ii
```

ND4

```
ND4 Syl.dna      1 TAGCTAAATGTTAAAAATATCAATAATAATAATAAATTTTGTAGTACTTATGTTAGTTATTATATATATAAACAT   75
ND4 CL.dna       1 TAGTTAAATGTTAAAAATAACAATAATAACAATAAATTTTGTAATGCTTATGTTAGTTATTATATATATAAACAT   75
ND4 Esmo.dna     1                                                                               0
                          ^^^
                                                             s             ss      s    s
ND4 Syl.dna     76 AAATTATAGTTTTTGTTTTGCAATAGAGATTAACTATATATATATAAATATGTATGTCAATTATATATCGCTTTG  150
ND4 CL.dna      76 AAATTATAGTTTTTGTTTTGCAATAGAAATTAACTATATATATATAAATATGTATGCTAATTATGTATCGCTTTG  150
ND4 Esmo.dna     1                                AACTAT-TATACATAAATATGTATGTCAATTATATATCACTTTG   52
                                                        i
                   s   s s              ss  s ss    ss       s   s    s     s  ss         ss
ND4 Syl.dna    151 ATTTATATTTTTAATGGGAATCATTCTGTTTTTCCTATTTTTCCTATTATCAAAAAAATGCATATCATATAATAA  225
ND4 CL.dna     151 ATTTGTGTTTTTAATGGGAATTGTTTTGCTTTTTCTATTTTTTCTATTATCAAAAAAATGTATATCATATAACAA  225
ND4 Esmo.dna    53 GTTTATATTTTTAATGGGAATTGTTTTATTTTTCTTATTTTTCCTAGTATCCAAAAAGTGTGTATCATATAGCAA  127

                   s        ss s   sss           s    s         s            s          s  sss
ND4 Syl.dna    226 ATATTTTTATGTGATATACATATACATATATATTTATATAAGTGTAATAACAATAATAATGATAGACGATTTTGC  300
ND4 CL.dna     226 GTATTTTTACGTGATACATATATACATATATATTTATATAAGTGTGATAACAATAATAATGATAGACGATTTCGT  300
ND4 Esmo.dna   128 ATATTTTTATATAATATCTATATACATATACATTTTTATAAGTGTAATAACAATAATAGTGATAGACGACTTTAT  202

                   s  s         s    s           s              s  s   s       s     s  s  s
ND4 Syl.dna    301 ATGTTTTATGATATTTTTCGAAAGTCTATTCTTCCCAATATGTATCGTAAGTTTATTTTTTAATTTTAACAACAG  375
ND4 CL.dna     301 ATGTTTTATGATACTTTTTGAAAGTCTATTTTTCCCAATATGTATGGTCAGTCTATTTTTTAATTTTAACAATAG  375
ND4 Esmo.dna   203 GTGCTTTATGATACTTTTTGAAAGTCTATTTTTCCCAATATGTATTGTAAGTTTATTTTTCAATTTCAATAACAG  277

                      s                 s     s     s  s               ss       s s
ND4 Syl.dna    376 ATTTATATTTGCAATATTTTACTTAGTAATTTTTAGCTCAATAAGTTCAATAGCATGTATAGTAATATGTATGAT  450
ND4 CL.dna     376 ATTCATATTTGCAATATTTTATTTAGTAATTTTCAGTTCAATAAGTTCAATAGCATGTATAGTAATATGTATGAT  450
ND4 Esmo.dna   278 ATTTATATTTGCAATATTTTATTTAGTCATTTTTAGTTCAATAAGTTCAATATTATGTATAATTATATGTATGAT  352

                    s       s            s       s                          s        s
ND4 Syl.dna    451 AGTAATATCACATTTCAATATATTAAATTTACAAATTTTTTTAGATATTTGTTTTTTCGACAGTATGTATTTAGC  525
ND4 CL.dna     451 AATAATATCACATTTCAATATATTAAATTTACAAATTTTTTTAGATATTTGTTTTTTTGACAGTATATATTTAGC  525
ND4 Esmo.dna   353 AATAATATCGCATTTCAATATACTAAATTTGCAAATTTTTTTAGATATTTGTTTTTTTGACAGTATGTATTTAGC  427

                   s             s s       ss    s     s  s     s        s  s  s  s      s
ND4 Syl.dna    526 TATTTTTATATGAATCTTTTTATTCGTAATGTTTTCTATAAAATACCCAATATGACCATTTCACGTGTGATTACC  600
ND4 CL.dna     526 AATTTTTATATGAATCCTTTTATTTGTAATGTTTTCTATTAAATATCCAATATGGCCCTTCCACGTGTGACTACC  600
ND4 Esmo.dna   428 TATTTTTATATGAACCCTTTTATTTATAATATTTTCCATCAAATACCCAATATGACCATTTCATGTGTGATTACC  502

                               s        s  s           s            s       s  s      s
ND4 Syl.dna    601 GGAAATGCATGTGGAAGTAAATACCGAAATGAGTGTAGTATTAGCAAGTGTAGTATTAAAAAATAGGTTTTTTTG  675
ND4 CL.dna     601 GGAAATGCATGTTGAAGTAAACACCGAAATGAGTGTTGTATTAGCAAGTGTAGTATTGAAAA-TAGGGTTTTTTG  674
ND4 Esmo.dna   503 GGAAATGCATGTTGAAGTAAATACTGAAATGAGTGTTGTATTAGCAAGTATAGTATTAAAGA-TAGGTTTTTTTG  576
                                                                                 i
                    s           ss s  s  s     s    s s                s           s ss  s
ND4 Syl.dna    676 GGGTATTTAAATTTTTGTTTATGTCATTTAACAGTCTATCATTATGATTTTTGGGTTTTGTAGACACTATTATTA  750
ND4 CL.dna     675 GAGTATTTAAATTCCTGTTCATGTCATTCAACAGTTTATCATTATGATTTTTGGGTTTTGTAGATACCATCATTA  749
ND4 Esmo.dna   577 GGGTATTTAAATTTCTATTCATATCATTCAACAATTTATCATTATGATTTTTAGGTTTTGTAGATAGTATTATTA  651

                     s          s           s  ss    s              s              s     s   s
ND4 Syl.dna    751 TGCTAGGAATAACATTATTATCTATATCTTTAGTATTTTTATCTGATTATAAAAAAATAATAGCGAATTGATCGA  825
ND4 CL.dna     750 TGTTAGGAATAACGTTATTATCTATCTCCTTAGTATTTTTATCTGATTACAAAAAAATAATAGCAAATTGATCGG  824
ND4 Esmo.dna   652 TGTTAGGAATAACATTATTATCTATATCTCTAGTTTTTTTATCTGATTATAAAAAAATAATAGCAAATTGGTCGG  726

                          s      s s   s     s  s s        s  s              s   s ss
ND4 Syl.dna    826 TAATACACACAGGTATTGCATTAATATTACTGTGACATAATGATCTTTTATATATAGGACTTCTCGTTATGTGTA  900
ND4 CL.dna     825 TAATACATACAGGTATTGCATTAATATTATTATGACATAACGACCTTTTATATATAGGACTTCTAATTATGTGTA  899
ND4 Esmo.dna   727 TAATACATACAGGTGTCGCACTAATACTATTGTGACATAATGACCTTTTATATATAGGGCTTTTAGTTATGTGTA  801

                     s           s       ss s  s     ss          s  s     s  s
ND4 Syl.dna    901 ATTTATCACATATTTTAAGTTCTGCATTTATGTTTATAACAATAGGATATATGTATGACAATTATGGTGTAAGAA  975
ND4 CL.dna     900 ATCTATCACATATTTTAAGTTCTGCCTTTATGTTTATAACAATAGGATACATGTATGACAATTATGGTGTAAGAA  974
ND4 Esmo.dna   802 ATTTATCACATATTCTAAGTTCATCATTCATGTTCGTAACAATAGGGTATATGTACGATAATTATGGTGTAAGAA  876

                    s  s         s s  s                   s         s   s                s
ND4 Syl.dna    976 TTTTTATATTAATGATCTCCTTTTTTGGTATAAGTATATGAAGTTCATTGTTTTTAGGATTATTTTTATTCAATA 1050
ND4 CL.dna     975 TTTTTATATTAATGGTCTCATTTTTTGGTATAAGTATATGAAGTTCATTGTTTCTAGGATTATTTTTATTCAATA 1049
ND4 Esmo.dna   877 TATTCATATTAATGATTTCATTTTTTGGTATAAGTATATAAAGTTCATTATTTTTAGGATTATTTTTATTTAATA  951
```

```
                       s  s                            s       s     s         s       s
ND4 Syl.dna   1051 TAGACTTTCCATTTATGTTACTATTTTACGTGGATATATTTATTTTATATGGCTTAATATCAGTATCATTTATAT 1125
ND4 CL.dna    1050 TAGATTTCCCATTTATGTTACTATTTTACGTGGACATATTTATTTTATATGGTTTAATATCAGTATCATTTATAT 1124
ND4 Esmo.dna   952 TAGATTTTCCATTTATGTTATTATTTTATGTGGATATATTTATTCTATATGGTTTAATATCAGTATCATTTATAT 1026

                    s   s    s     s s             s                s               s    s
ND4 Syl.dna   1126 ATATTGTATGTTTTTACATAATTGTATTATCTATATTTATATCATCTATCTATATTTATATGTGTCTATCATTTT 1200
ND4 CL.dna    1125 ACATTGTATGCTTTTATATAATTGTATTATCTGTATTTATATCATCTATCTATATTTATATGTGTTTATCTTTTT 1199
ND4 Esmo.dna  1027 ACATTATATGTTTTTATACAATTGTATTATCTGTATTTATATCATCTATTTATATTTATATGTGTCTATCATTTT 1101

                       s     s  ss s     s   s     s s  ss    s     s  s     s           s   s
ND4 Syl.dna   1201 ATTCTTTTATTTGACTCGATAAGTATCTGCGTCTGGATTTAACGATAAACGACATATATTTTTATTTAGTGATAA 1275
ND4 CL.dna    1200 ATTCTTTTATCTGATTAGATAAATATCTGCGTTTGGATCTAACAATAAATGATATATATTTTTATTTAGTAATAT 1274
ND4 Esmo.dna  1102 ATTCGTTTATATGGCTTGATAAATATTTGCGTTTAGACTTAACAATAAACGATATATACTTTTATTTAGTAATAG 1176

                       ss    s
ND4 Syl.dna   1276 CAACGATACTTATAATTTTTTATTATGTCATATATTTATTATTTTAAAATT 1326
ND4 CL.dna    1275 CAACGATACTTATAATTTTTTATTATGTCATATATTTATTATTTTAAGAAT 1325
ND4 Esmo.dna  1177 CAACAGTACTCATAATTTTTTATTATGTCATATATTTATTATTTTAAAAAT 1227
                                                               ^^^
```

ND3 unedited

```
                   s   s          s
AGAATCACGAGTTGAATTCAAGAAGGATTTTTGGGGGAGACTTTTTTA CCAA       Sylvio DNA
AGAATCACGAGTTGAATTCAAGAGGGA TTTTGGAGGAGAC TTTTTA CCAA       CL Brener DNA
AGAATCACGAGTTGAATTCGAGAAGGA TTTTGGAGGAGA   TTTTATCCAA       Esmeraldo DNA
                           i            iii     i

          s                     ss
GGGTTAA TAGTTTATTTATATTTTGAGAGAAGGTTTTGGTTTGAAGGGAA
GGGTTAA TAGTTTATTTATATTTTGAGAGAAGATTTTGGTTTGAAGGGAA
GGG  AATTAATTTATTTATATTTTGAGAGAAAATTTTGGTTTGAAGGGAA
   ii  i

             s                       s
 GTTGGTTG TTTAGGAGG TGACCA TTTGA TTTTATTTTGTTTGG TAAC
 GTTGG TG TTTAGGAGGTTGACCATTTTGATTTTTATTTTGTTTGG TAAC
TG  GGTTGTTTTGGGAGGTTGACCA TTTGA TTTTGTTTTGTTTGGTTAAC
i ii  i  i         i      i     i               i

    s ss  ss    ss           ss    s
TTAATGGATTAATTTTAGAGTATTTTGTTTGAA TTTTAAAATAA
TTAATGAG TGGTTTTGAAGTATTTTGTTTGAATTCTTAAAATAA
TTAACGGA TAA   TAAAGTATTTTGTTCAAATTTTTAAAATAA
        i   iii
```

RPS12

```
A GTAAATATATTTTGTTTTTTTTGCGTATGTGA TTTTTGTATG   GTTGTTGTTTAC    GTTTTGTTTTATTTGTTTTA   Tb edited
                        s
A A AAA A A   TG        ACG A G GATTTTTTG A GTTTG  G  G TTRCTTTTGTTT G    G   G    A   Sylvio DNA
A A AAA A A   TG        GCG A G GATTTTTTG A GTTTG  G  G TTACTTTTGTTT G    G   G    A   CL Brener DNA
ATA AAA A A   TG        GCG A G GATTTTTTG A GTTTG  G  G TTACTTTTGTTT G    G   G    A   Esmeraldo DNA
 i

TGTTATTATATGAGTCCGC    GATTGCCCAG TTCCGGTAACCGACGTGTATTGTATGC  C  GTATTTTA TTTATATAAT

 G  A  A A GAGCCCACTTTTGA  ACCCAG  TCCGG AACCGTCG G G  A A GCTTCTTG A    ATTTTG A AG
 G  A  A A GAGCCCAC TTTGA  ACCCAGTTTCCGG AACCGTCG G G  A A GCTTCTTG A    ATTTTG A AG
 G  A  A A GAGCCCAC  TTGA  ACCCAG TTCCGG AACCGTCG G G  A A GCTTCTTG A    ATTTTG A AG
                   ii             ii

TTTGTTTGGATGTTGCG TTGTTTTTTTTGTTGTTTTATTGGTTTAGTTATG  TCATTATTTATTATAGA G   G  GTGGT

   G   GGG G  GCG TTG        G  G    G  AA    AGC A GTTTCA  A   A  A AGATA TTGTTG GG
   G   GGG G  GCGTTTG        G  G    G  AA    AGC A GTTTCA  A   A  A AGATA TTGTTG GG
   G   GGG G  GCG TTG        G  G    G  AA    AGC A GTTTCA  A   A  A AGATATTTGTTG GG
                 i                                                       i

GGTTTTGTTGATTTACCC      G    GTGTAAAGTATTA TACA CGTATTG   TAAGTTAG
                                                                s
GG    A  GA    ACCC TTTTTGTTTTG ATAAAA A  ATTACATCGTA  GTTTTAAGTTGA
GG    A  GA    ACCCTTTTTTGTTTTG ATAAAA A  ATTACATCGTA  GTTTTAAG TGA
GG    A  GA    ACCC  TTTTGTTTTG ATAAAA A  ATTACATCGTA  G TTTAAGTTAA
                  ii                                   i      i
```

ND5

```
                   s s                  s          ss    v         v s  s  s
   1 TAAAGATGTTTTTGCTATTTTTTGCAATTTTTTTTCTGTTTGGTTTAATATCCGGAACTATATTATTAGGTAGGC 75
   1   AAGATGTTTTTGTTGTTTTTTGCAATTTTTTTTCTGTTTGGTTTGATATCAGGAACTATAATGTTGGGCAGGC 73
   1  TAAAATGTTTTTGTTGTTTTTTGCAATTTTTTTTTTGTTTGGTTTAGTATCAGGAACTATAATGTTAGGTAGGC 74

                               s s  s    sv           s   s  s    s         ss
  76 ATTTTATAAGTTTTTGACAATCAATTGTATTAAGTATTTTTATATTATTATGTATAATATTTACTTATTATATAA 150
  74 ATTTTATAAGTTTTTGACAATCAATTATGTTGAGTACTTTTATATTATTATGTGTAGTATTTACTTATTATGTAA 148
  75 ATTTTATAAGTTTTTGACAATCAATTATATTAAGTACATTTATATTATTGTGTATAGTATTCACTTATTATGCAA 149

                        s     s     s  s     s                         v s    ss
 151 TTGGTGTATGTTTATATGGATATTGTTATTATGACTTTTGCTTAATATTAATGTTAGATTTTTGTTTTATATGGC 225
 149 TTGGTGTATGTTTATATGGGTATTGTTATTACGATTTTTGTTTAATATTAATGTTAGATTTTTGTTTTGTATGAC 223
 150 TTGGTGTATGTTTATATGGATATTGCTATTACGATTTTTGTTTAATATTAATGTTAGATTTTTGTTATATATGAT 224

         s        s  s  v  s        s   s s     s      s          ss v   s    s
 226 TAACCTTTTCATGTTCAGGTTTCTATTTGTACATACTGTTGTTGATAGATCTTGTATTTTGTTTTATACTATTTT 300
 224 TAACTTTTTCATGCTCAGGATTTTATTTGTACATATTGTTGTTGATAGATCTTGTATTTTGCTTTATATTATTTT 298
 225 TAACTTTTTCATGCTCGGGGTTCTATTTGTATATATTATTGTTAATAGATTTTGTATTTTGTCTAATATTATTCT 299

            s     s        s              s              v           ss    s
 301 ACGCATTTTATTACATGTATTTTGATATATTTTTAGGGCGATTTCTTAATATTTTTTGAATGTTCATTTTATGTA 375
 299 ACGCATTTTATTATATGTATTTTGATATATTTTTAGGACGATTTCTTAATATATTTTGAATGTTTGTTTTATGTA 373
 300 ACGCATTCTATTATATGTATTTCGATATATTTTTAGGGCGATTTCTTAATATTTTTTGAATGTTTGTTTTGTGTA 374

               s                       v  v           s           s
 376 TGAATTTTTCATATTATCATATGATTTTATAACAGCATATTGTGGTTGAGAATTATTAGGATTATATTCTTTTT 450
 374 TGAATTTTTTTATATTATCATATGATTTTATAACCGCTTATTGTGGTTGGGAATTATTAGGATTATATTCTTTTT 448
 375 TGAATTTTTTTATATTATCATATGATTTTATAACTGCCTATTGTGGTTGGGAATTATTAGGGTTATATTCTTTTT 449

      vvvs     s           s     sv s s
 451 TATTAATATCTTATTTTTGATACCGTTTCATCTCC-ATAAAATTCGGTTTTAAAGCATTTTTTATAGGTAAAATA 524
 449 TTTTGATATCTTATTTTTGATATCGTTTTATTTCC-ATAAAATTCGGTTTTAAAGCATTTTTTATAGGTAAAATA 522
 450 TTAAAATATCCTATTTTTGATACCGTTTTTTTTTCTATCAAATTCGGTTTTAAAGCATTTTTTATAGGTAAAATA 524
                                        i
           s v        v            s  s s              v     sss      s        v
 525 GGAGATATATTGCTTATATTTGCATTTTCAATACTATTTTCAATGAATGGTTATTGCACAGCAACATTTCATATT 599
 523 GGAGATATCTTGCTTATCTTTGCATTTTCAGTATTATTTTCAATGAATGGTTATTGTATAGCAACGTTTCATATA 597
 525 GGAGATGTATT-CTTATATTTGCATTTTCAATATTGTTTTCAATGAATGGATATTGTGTAGCAACATTTCATATT 598
                i
       v  s   v s     s  s  s     s    s  v             s  v                s  s
 600 ATAAACTTTATGTGTGTTGATTACTATTACATAGTATTTATAACTTTAATATTATTATGTTGTGCTTTTACGAAA 674
 598 ATTAATTTTTTGTGTGTTGACTACTATTATATAGCATTTATAACTTTAATATTATTATGTTGTGCTTTTACAAAG 672
 599 ATAAACTTTTTATGTGTCGATTATTATTATATAGCATATATAACTTTAATACTAGTATGTTGTGCTTTTACAAAA 673

                   ss s  s        s  s     s                    v
 675 AGTACACAGTTTGGGCTACATATTTGATTACCAGATGCAATGGAAGGACCAATACCTGTATCTGCATTAATACAT 749
 673 AGTACACAGTTTGGGTTGCATATTTGATTGCCAGATGCAATGGAAGGACCAATACCTGTATCTGCATTAATACAT 747
 674 AGTACACAGTTTGGATTACACATTTGATTACCGGATGCGATGGAAGGACCAATACCTGTCTCTGCATTAATACAT 748

          v                                   ss       s     s
 750 GCTGCAACGTTAGTTGTATGTGGAATAATATTAGGTAGTTTCATATTTTGGTGTTTCGAATTTTGATTTAGCTAT 824
 748 GCTGCAACGTTAGTTGTATGTGGAATAATATTAGGTAGTTTTGTATTTTGGTGTTTTGAATTTTGATTTAGCTAT 822
 749 GCTGCCACGTTAGTTGTATGTGGAATAATATTAGGTAGTTTTGTATTTTGATGTTTTGAATTTTGATTTAGCTAT 823

              v       s  s   sss v         v           s     s    s      s    ss
 825 TTTTATAGTATAATTTGCTGAGCAACCGCAATAATAGTATTAATGAGTCTGTGTGTCTTTTGTAATTTTGACGCA 899
 823 TTTTATAGTCTAATTTGTTGGGCAACCGCAATAATAGTTTTAATGAGTCTGTGTGTTTTTTTATAATTTCGACGCA 897
 824 TTTTATAGTATAATTTGTTGAGCAGTTGGAATAATAGTGTTAATGAGTCTATGTGTTTTTTTATAATTTCGACGTG 898

       s  v        s           v  s              s        s   v    s
 900 AAACGTTATATAGCATTTAGTACAATCTGCCAAATAAGTTTTTCGTTTTTTTGCTGTATTTGCTTAGATCTTTAT 974
 898 AAGCGTTATATAGCGTTTAGTACAATATGTCAAATAAGTTTTTCATTTTTTTGTTGTCTTTGTTTAGATCTTTAT 972
 899 AAACGGTATATAGCATTTAGTACAATATGTCAAATAAGTTTTTCATTTTTTTGTTGTCTTTGTTTAGATCTTTAT 973

     v    s   s                s        s        v                 vs
 975 GTAGGTTGTTTATTTTTTTGTTATCACATGTTTTATAAGGCATCTTTGTTTATAGTAATAGGTGTATGAATACAT 1049
 973 GTAGGCTGTCTATTTTTTTGTTATCACATGTTTTATAAGGCATCATTGTTTATAGTAATAGGAATATGAATACAT 1047
 974 CTAGGCTGTCTATTTTTTTGTTATCATATGTTTTACAAGGCATCATTGTTTATAGTAATAGGAATATGAATACAT 1048

          s      s    s   s s                    s              s  s           s
1050 ATATTCTTTGGTCTACAAGATCTAAGATGTTATTTTTTTATATACTTTTGTGGTTGTATTTTAGCAAGATTTTTA 1124
1048 ATATTTTTTGGTTTACAAGATTTGAGATGTTATTTTTTTATATATTTTTGTGGTTGTATCTTGGCAAGATTTTTA 1122
1049 ATATTTTTTGGTTTACAGGATTTAAGATGTTATTTTTTTATATATTTTTGTGGTTGTATTTTAGCAAGATTTTTG 1123
```

ND5 p.2

```
             s  s   s    s  v                       v        s  s      s       s
1125 TTAATATTCGCTATATTAAACTCATGTTCAATTTGATTTTTGTGTGGATTTTATTGCAAGGATATGTTATTAAGT 1199
1123 TTAATATTTGCCATATTAAATTCATGTTCAATTTGATTTTTGTGTGGTTTTTATTGTAAAGATATGCTATTAAGT 1197
1124 TTAATATTTGCTATACTAAACTCTTGTTCAATTTGATTTTTGTGTGGTTTTTATTGTAAGGATATGTTATTAAGC 1198

           s s       ss     s                       s  ss  s s  s        v
1200 ATGTTGTTGACATTAGCCTTTTACAATATTATAGAATTTTTGGTTGTGTGTATCATATTTATATTTTTAACAATA 1274
1198 ATGTTGTTGACATTAGCCTTTTATAATATTATAGAATTTTTGGTTGTATGCATCATGTTCATATTTTTTACAATA 1272
1199 ATGTTGCTAACATTA-TTTTTTACAATATTATAGAATTTTTGGTTGTATGTGTCGTATTTATATTTTTTACAATA 1272
                    i
     s             ss    s  s  ss       s        s  s     s  s        ss
1275 ATATACAATTATTTCTTATTGTTCTTCCTATTTTTCGTGTTTAAATGCTTTTGTTTAGTAGATTGTTTATTTTTA 1349
1273 ATATACAATTATTTCTTATTGTTTTTCTTATTTTTCGTGTTTAAATGTTTTTGTTTGGTAGATTGCCTATTTTTA 1347
1273 GTATACAATTATTTTCTATTATTTTTTCTATTTTTTGTGTTTAAGTGTTTTTGCTTAGTAGATTGTTTATTTTTA 1347

       s  s               v ss         s         v        s
1350 TTATTCGATTTTGAATGTTGTATCATTTATTGTATGTTATGTGTGTATATGTGCTTTATATGTATATTTTTTGTG 1424
1348 TTATTTGATTTTGAATGTTGTCTTGTTTATTGTACGTTATGTGTTTATATGTGTTTTATATGTATATTTTTTGTG 1422
1348 TTGTTTGATTTTGAATGTTGTCTTGTTTATTGTATGTTATGTGTGTATATGTGTTTTATATGTATATTTTTTGTG 1422

       s  s   s    s           s        s  v ss     sss   s         s    s
1425 TTAGATTATGTTTATACATTTATATACGCAAGTTACACAATGTT---AACATTCTATTTATATTTTTACAGTTTT 1496
1423 TTGGACTATGTTTACACATTTATATATGCAAGTTATACTACATT---GGTATTTTATTTATATTTTTACAGTTTT 1494
1423 TTGGACTATATTTATACATTTATATACGCAAGTTACACTATGTTCTTAGTATTTTATTTATATCTTTATAGTTTT 1497
                                                 iii
       s     s        s         s     s  v s v         s     s    ss ss
1497 TTTGATATCACTATATTAGTATTGTTTGTAATAATATTATCTGCATTTATATATTACGGAAATATCTTTTTTTAT 1571
1495 TTCGATATTACTATATTAGTATTGTTTGTAATAGTAATGTTTGCATTTATATATTATGGAAGCACTTTTTTTTAT 1569
1498 TTTGATATTACTATATTGGTATTGTTTATAATAATAATGTGTGCATTTATGTATTATGGAAGTACTTTTTTT-AT 1571
                                                                             i
               s                vs
1572 TTTTTTAATACGGATGTTGTTATGTTATTTTGAA                                          1605
1570 TTTTTTAATACGGATGTTGTTATGTTATTTTGAAGAATATTTCTAATTATAGTAACATTATTTATATTTTCAATA 1644
1572 TTTTTTAATATGGATGTTGTTATGTTAGCTTGAAGAGTATTTTTAATTATAATAACACTGTTTATATTTTCGATA 1646
```
